# A *Lacticaseibacillus rhamnosus* secretome induces immunoregulatory transcriptional, functional and immunometabolic signatures in human THP-1 monocytes

**DOI:** 10.1038/s41598-024-56420-8

**Published:** 2024-04-10

**Authors:** Michael P. Jeffrey, Lin Saleem, Chad W. MacPherson, Thomas A. Tompkins, Sandra T. Clarke, Julia M. Green-Johnson

**Affiliations:** 1 Applied Bioscience Graduate Program and the Faculty of Science, Ontario Tech University, Oshawa, ON L1G 0C5 Canada; 2 Lady Davis Institute for Medical Research, Jewish General Hospital, Montreal, QC H3T 1E2 Canada; 3 Lallemand Bio-Ingredients, Inc., Montreal, QC H1W 2N8 Canada; 4 Guelph Research and Development Centre, Agriculture and Agri-Food Canada, Guelph, ON N1G 5C9 Canada

**Keywords:** Microbiology, Immunology, Cytokines

## Abstract

Macrophage responses to activation are fluid and dynamic in their ability to respond appropriately to challenges, a role integral to host defence. While bacteria can influence macrophage differentiation and polarization into pro-inflammatory and alternatively activated phenotypes through direct interactions, many questions surround indirect communication mechanisms mediated through secretomes derived from gut bacteria, such as lactobacilli. We examined effects of secretome-mediated conditioning on THP-1 human monocytes, focusing on the ability of the *Lacticaseibacillus rhamnosus* R0011 secretome (LrS) to drive macrophage differentiation and polarization and prime immune responses to subsequent challenge with lipopolysaccharide (LPS). Genome-wide transcriptional profiling revealed increased M2-associated gene transcription in response to LrS conditioning in THP-1 cells. Cytokine and chemokine profiling confirmed these results, indicating increased M2-associated chemokine and cytokine production (IL-1Ra, IL-10). These cells had increased cell-surface marker expression of CD11b, CD86, and CX_3_CR1, coupled with reduced expression of the M1 macrophage-associated marker CD64. Mitochondrial substrate utilization assays indicated diminished reliance on glycolytic substrates, coupled with increased utilization of citric acid cycle intermediates, characteristics of functional M2 activity. LPS challenge of LrS-conditioned THP-1s revealed heightened responsiveness, indicative of innate immune priming. Resting stage THP-1 macrophages co-conditioned with LrS and retinoic acid also displayed an immunoregulatory phenotype with expression of CD83, CD11c and CD103 and production of regulatory cytokines. Secretome-mediated conditioning of macrophages into an immunoregulatory phenotype is an uncharacterized and potentially important route through which lactic acid bacteria and the gut microbiota may train and shape innate immunity at the gut-mucosal interface.

## Introduction

Antigen-presenting cells (APC), such as macrophages and dendritic cells, participate in antigen processing and cytokine production, facilitating innate and adaptive immune interactions^1^. Typically, macrophage activity varies with phenotype, with macrophages polarized to the M1, or classically activated phenotype, showing pro-inflammatory activities (increased phagocytic capacities and secretion of inflammatory mediators). In contrast, polarized M2 or alternatively activated macrophages are associated with immunoregulatory activity (tissue remodeling and secretion of immunoregulatory mediators), with current models reflecting a range of phenotypes within these categories^2–4^. These polarization states remain fluid and adaptive to macrophage microenvironments and in the small intestine, where the mucin layer covering intestinal epithelial cells (IEC) is porous, the sampling of antigens and contact with microbial components and products by the underlying APC cell populations helps to shape host immune outcomes. Consequences of these host-microbe interactions include certain epigenetic and immunometabolic modifications of APC associated with innate immune memory in the form of innate immune tolerance and trained innate immunity^5–8^. For example, initial contact of myeloid cells with β-glucan, a fungal structural component, induces persistent epigenetic reprogramming, which primes the innate immune system to respond more robustly to subsequent challenge via increased trimethylation of H3K4^9^.

Within gut-associated lymphoid tissues, a range of APC phenotypes act in order to maintain immune homeostasis yet remain responsive to pathogens, reacting to many microbial signals through pattern recognition receptor (PRR) activation. However, since the mucosal surface is covered with a mucin layer that can impede direct contact with gut microbes, secretion of biologically active mediators able to pass through the mucin layer and interact directly with IEC and APC may be an important route through which lactic acid bacteria (LAB), and potentially other gut microbes, influence the immune system at the mucosal interface. While certain LAB can polarize differentiated macrophages^10^, many questions surround the mechanisms involved and the role of LAB and their secreted molecules in the differentiation and subsequent polarization into immunoregulatory APCs through transcriptional and immunometabolic reprogramming.

Mechanistic evidence provides insight into the capacity of certain LAB for immune modulation and their impact on host immune outcomes^11–14^. While the ability of LAB to influence macrophage activity has been reported^15,16^, little is known about the roles of postbiotic secretome components derived from LAB in the transcriptional and functional reprogramming of macrophages, a potentially important route for gut microbes to influence host immune activity. Lactobacilli secretomes are complex, containing a range of bioactive and immunomodulatory molecules, including peptidoglycan components (LTA), peptides, indole derivatives and a range of other metabolites^17,18^. Secretome-mediated immunomodulatory activity of these bacteria is also of current interest in the context of postbiotics, which includes cell free microbial metabolites and components. Our recent transcriptomic analysis revealed context-dependent regulation of tumor necrosis factor α (TNFα) and *Salmonella enterica* subsp. *enterica* serovar Typhimurium secretome-induced pro-inflammatory mediator transcription and production by the *Lacticaseibacillus rhamnosus* R0011 (formerly *Lactobacillus rhamnosus* R0011) secretome (LrS) in human IEC, indicating a potential for postbiotic modulation of pro-inflammatory immune activity with minimal IEC impact in the absence of a pro-inflammatory challenge. Our findings indicate that this modulation is mediated through induction of negative regulators of innate immunity and through changes in global histone acetylation patterns^13^, events important in maintaining immune regulation. We have also found that *L. rhamnosus* R0011 cultured in milk can induce a regulatory phenotype in lipopolysaccharide (LPS)-challenged PMA-differentiated THP-1 macrophages^19^.

In the current study, we aimed to delineate the impacts of LrS on macrophage activity by investigating the temporal transcriptional and functional reprogramming of macrophage phenotype and on responses to LPS challenge, using THP-1 human monocytes. THP-1 monocytes are a well-established model for studying monocyte and macrophage activity in vitro and can readily be differentiated into macrophages with characteristics of M1 or M2 phenotypes^20,21^. LPS challenge of THP-1 monocytes results in a phenotype and transcriptional profile shared with LPS-challenged peripheral blood mononuclear cell-derived macrophages isolated from healthy donors^22,23^, making THP-1s a useful cell model to study the impacts of LPS challenge and LrS conditioning on human monocyte and macrophage activity. THP-1 monocytes can also be differentiated into a resting macrophage phenotype, providing a useful model to examine LrS impacts on macrophages in combination with retinoic acid (RA), a key immunoregulatory mediator in the gut mucosal environment^24^. The aim of the present study was to examine functional and transcriptional reprogramming of macrophage activity by secretome components derived from LAB. Using genome-wide transcriptional profiling, cytokine/chemokine production analysis, cell-surface protein expression and mitochondrial substrate utilization assays, the data presented here provides evidence supporting the potential for gut microbial secretome-mediated functional re-programming of macrophage activity.

## Results

### LrS conditioning induces differential and temporal gene expression profiles in THP-1 human monocytes

**Figure 1:**
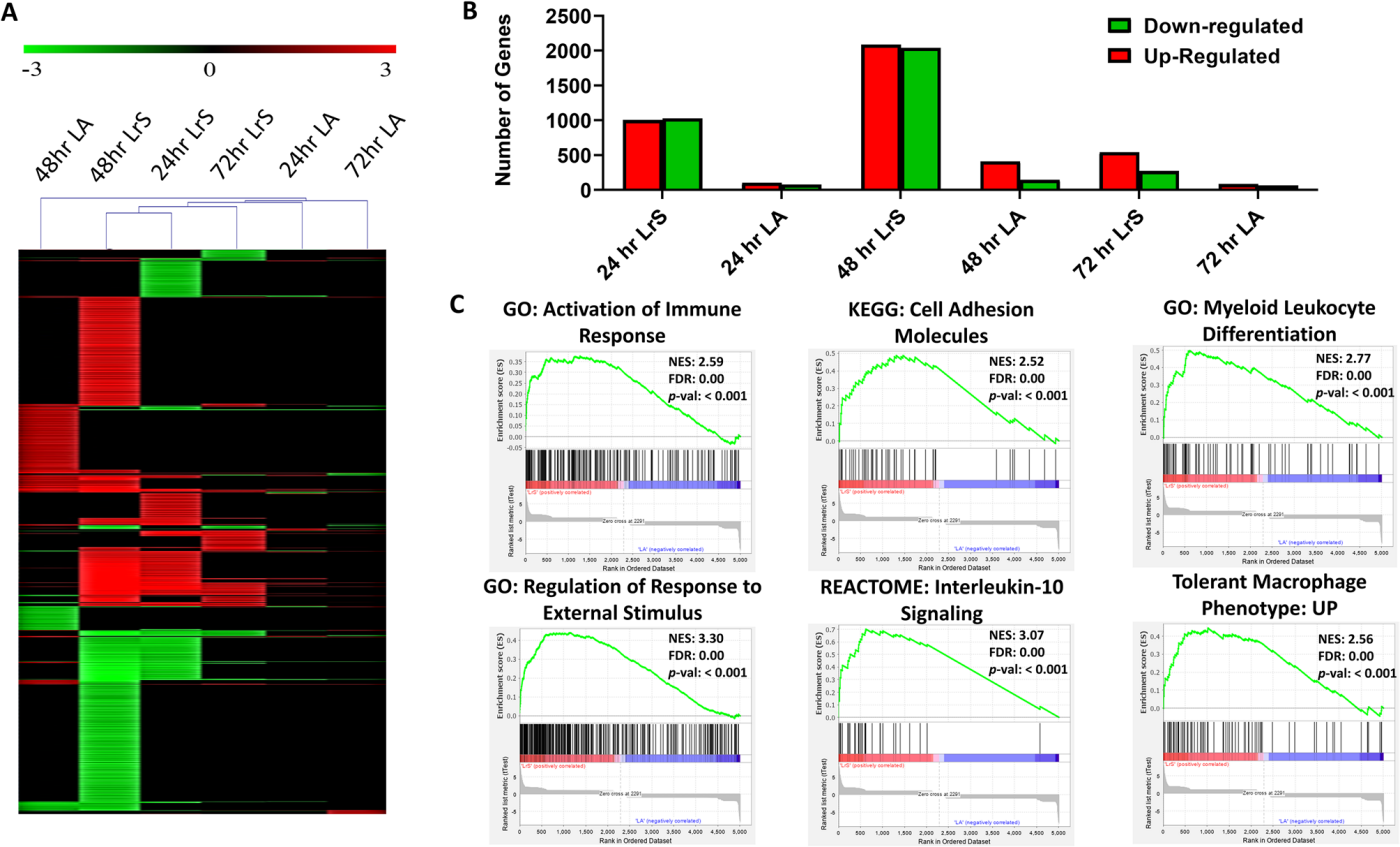
The LrS induces unique temporal transcriptional profiles in THP-1 human monocytes distinct from L-LA controls. (**A**) Two-dimensional hierarchical clustering analysis of global gene expression patterns in THP-1 human monocytes conditioned with the LrS or LA-matched controls for 24 h, 48 h, or 72 h (*n* = 4; *p* < 0.05; fold change different > 1.5 vs. media-control treated cells). (**B**) Total number of up- and down-regulated genes in response to each of the different challenges. (**C**) Gene set enrichment analysis (GSEA) revealed the enrichment of key macrophage signaling pathways activated by conditioning with the LrS (red) when compared to LA-matched controls (blue) as determined by the ranked t-test list metric over all tested time points. The green line represents the enrichment score and the black lines indicate where the genes within the gene set fall within the ranked pathway gene list.

Genome-wide transcriptional profiling of THP-1 human monocytes cultured with the LrS or LA matched controls was performed to evaluate global changes in the THP-1 transcriptome in response to LrS conditioning. Two-dimensional hierarchical heat-map cluster analysis revealed that conditioning THP-1s with the LrS resulted in unique temporal gene expression profiles, distinct from LA matched controls. In fact, LA had limited and unique impacts on THP-1 expression profiles (Fig. 1A,B) indicating that although this bioactive molecule is a key metabolite in the LrS, it is not responsible for the LrS-mediated impact on transcriptional activity. 48 h of LrS conditioning had the largest impact on the THP-1 transcriptome, with a total of 4126 differentially expressed genes (2086 up-regulated and 2040 down-regulated) (*n* = 4, *p* < 0.05). Of the three different time points tested, 72 h of conditioning with the LrS had the lowest impact on the THP-1 transcriptome, with only 813 differentially expressed genes (542 up-regulated and 271 down-regulated) (*n* = 4, *p* < 0.05), indicating a distinct temporal effect of LrS conditioning. (Fig. 1B).

To interrogate the cellular pathways impacted in THP-1 monocytes following LrS conditioning, gene enrichment analysis was performed using GSEA and gProfiler. GSEA analysis revealed that the LrS induced differential expression of genes involved in key macrophage signaling pathways such as the activation of the immune response (Normalized Enrichment Score (NES) = 2.59, *p* < 0.001), cellular responses to external stimulus (NES = 3.30, *p* < 0.001), cell adhesion molecule expression (NES = 2.52, *p* < 0.001), and the up-regulation of genes associated with interleukin-10 signaling, myeloid leukocyte differentiation, and a tolerant macrophage phenotype (NES = 2.56, *p* < 0.001) when compared to cells treated with LA controls (Fig. 1C).

### The LrS polarizes THP-1 monocytes into immunoregulatory M2 macrophages

**Figure 2:**
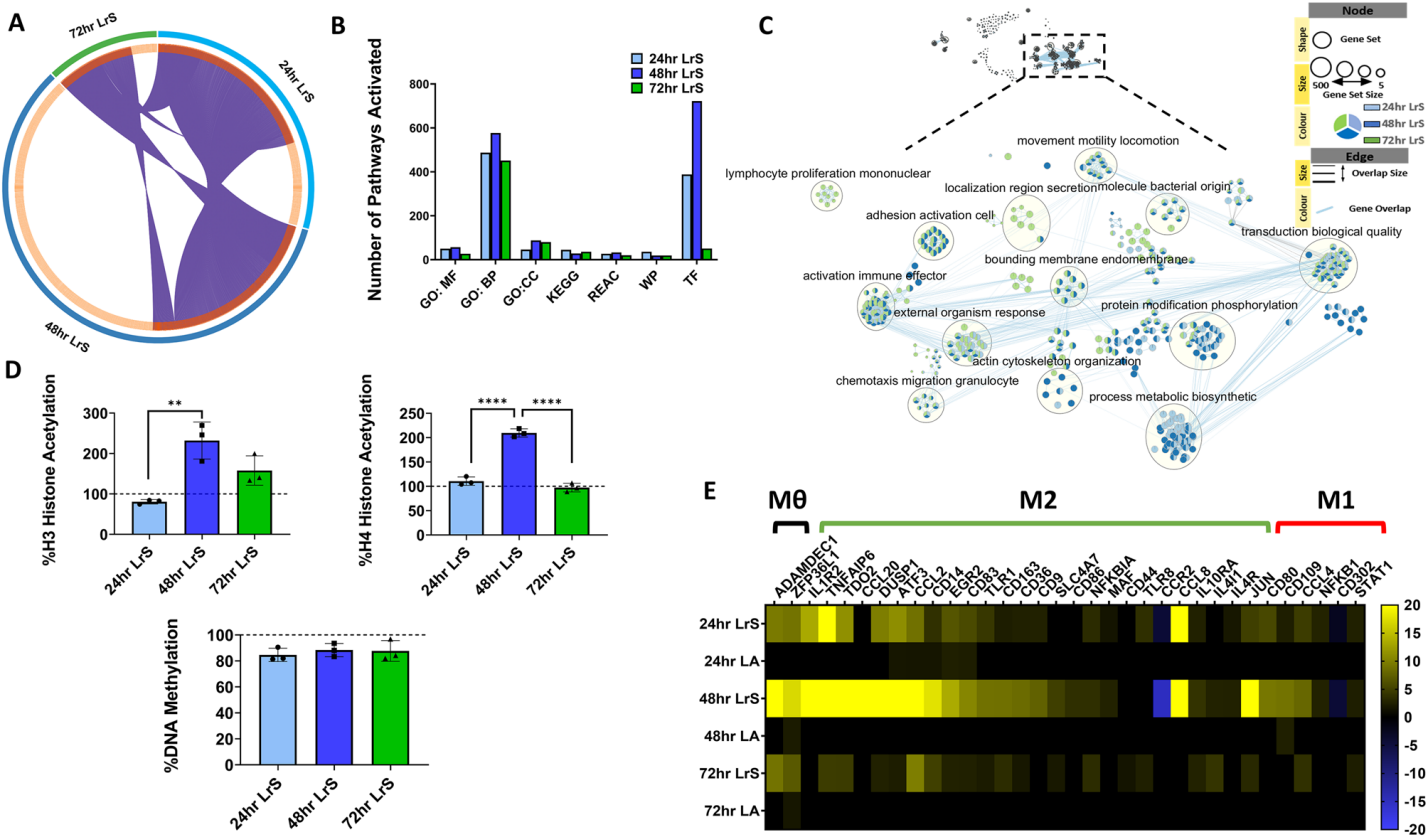
(**A**) Circos Plot analysis identified unique temporal gene signatures in THP-1 monocytes conditioned with the LrS. (**B**) gProfiler gene enrichment analysis revealed the activation of many key signaling pathways by the LrS in THP-1 monocytes (*MF* molecular function; *BP* biological process; *REAC* Reactome pathways; *WP* wikiPathways; *TF* transcription factors). (**C**) Visualization of the differential activation of key cellular pathways identified by gProfiler by EnrichmentMap. (**D**) Changes in global histone H3 or H4 acetylation patterns and DNA methylation in THP-1 human monocytes conditioned with the LrS. Data shown are the mean change in percentage of acetylation or DNA methylation compared with untreated controls ± SEM (n = 3). Significance is indicated as *p < 0.05, **p < 0.01, ***p < 0.001 as determined by one-way ANOVA and Tukey’s post-hoc test. (**E**) THP-1 human monocytes conditioned with the LrS show similar transcription profiles to those seen in immunoregulatory macrophage activation (*n* = 4; *p* < 0.05; fold change different > 1.5 vs. untreated cells).

Although there were unique differential gene expression profiles induced by the LrS over the different time points (Fig. 2A), gProfiler analysis identified a high degree of functional similarity of differential gene expression profiles and pathway activation between the different LrS conditioning time points, with most of the differential pathway activation occurring following 48h of conditioning (Fig. 2B). EnrichmentMap visualization of activated functionally related pathways identified by gProfiler revealed overlap between the different LrS conditioning time-points with many of the activated cellular pathways belonging to cell cycle, transcription, metabolism, and the cellular response to molecules of bacterial origin (Fig. 2C), confirming the results obtained by the GSEA analysis. Moreover, genes belonging to functional groups relating to cytoskeletal reorganization and metabolic and biosynthetic processes were largely influenced by 48h of LrS conditioning, indicating a significant impact on key macrophage cellular pathways (Fig. 2C). Levels of global H3 and H4 acetylation confirmed these findings, with the LrS significantly increasing histone acetylation percentages (%H3 and %H4) compared to untreated controls following 48h of conditioning (*n* = 3; *p* < 0.05) (Fig. 2D). However, the LrS did not have an impact on levels of methylated DNA relative to controls or between the three different time points (*n* = 3; *p* > 0.05) (Fig. 2D), suggesting unique transcriptional regulation induced by conditioning with the LrS.

Since GSEA analysis indicated that THP-1 monocytes conditioned with the LrS share gene expression signatures consistent with a tolerant macrophage phenotype and gProfiler analysis identified activated cellular pathways belonging to cell differentiation and metabolism (Fig. 1C), gene expression profiles were probed for increased transcription of genes associated with immunoregulatory macrophage activity. The LrS induced increased transcription of *ADAMDEC1*, *ZFP36L1*, and *RND3*, genes and transcription factors involved in differentiation of monocytes into mature macrophages (*n* = 4; *p* < 0.05). Temporal changes in gene expression of a wide range of genes associated with an immunoregulatory macrophage transcriptional signature (*IL-10R*, *TDO2, CD36, CD163, TLR1, TLR8, DUSP1,* and *ATF3*) was also observed following LrS conditioning, suggesting that the LrS polarizes THP-1 monocytes into an immunoregulatory phenotype, an effect independent of lactic acid (*n* = 4; *p* < 0.05). *IL1R2* (Interleukin 1 Receptor Type 2) was also highly up-regulated in THP-1s following 24- and 48 h of LrS conditioning (13.18 and 78.47 fold change respectively) (*n* = 4; *p* < 0.05) (Fig. 2E).

**Figure 3:**
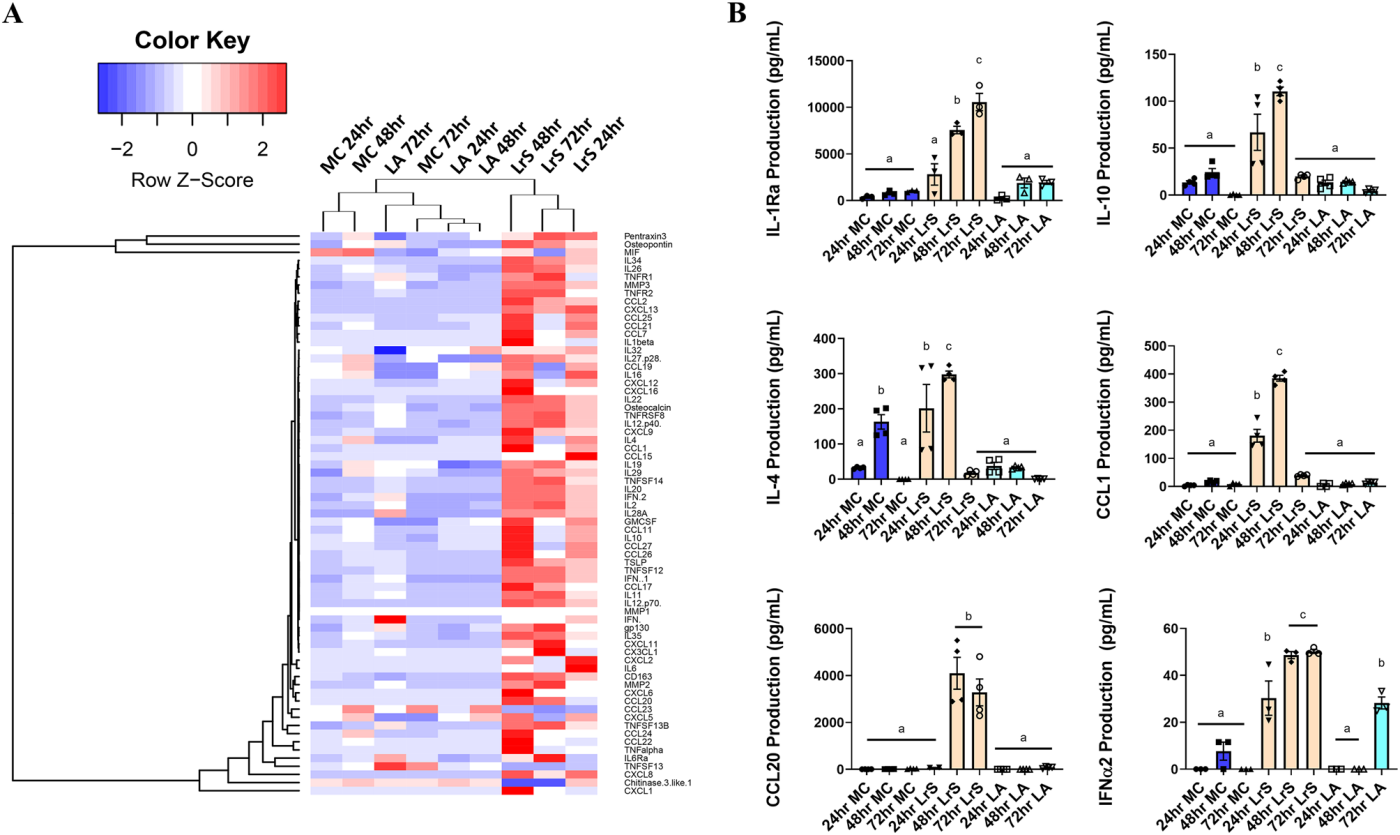
LrS conditioning of THP-1 human monocytes results in unique temporal cytokine and chemokine production profiles. (**A**) Two-dimensional hierarchical clustering analysis of cytokine and chemokine production profiles from THP-1 human monocytes following conditioning with the LrS or LA-matched controls for 24 h, 48 h, or 72 h. Data shown is the Z-score statistic for each cytokine and chemokine measured (*n* = 4). (**B**) The LrS induces the production of key cytokines and chemokines from THP-1 human monocytes (IL-1Ra, IL-10, IL-4, CCL1, CCL20, IFNα2). Data shown is the mean cytokine or chemokine production (pg/mL) ± SEM (n = 4). Statistical significance is indicated as ***p < 0.001 or ****p < 0.0001 as determined by Tukey’s one-way ANOVA.

Cytokine and chemokine profiling confirmed these findings, with increased temporal production of immunoregulatory macrophage-associated mediators IL-1Ra, IL-10, CCL1, CCL17, CCL20, CXCL1 and CXCL2, IL-6, IL-4 and MMP-2 following conditioning with the LrS, an effect independent of LA (Fig. 3; Figs. S1 and S2). There was also an increase in the production of some classical pro-inflammatory cytokines and chemokines following 48 h of LrS conditioning, but not after 72 h (Fig. 3; Figs. S1 and S2). This is similar to the results seen with transcriptional profiling and suggests that prolonged exposure to the LrS induces a tolerant immunoregulatory phenotype in THP-1 human monocytes.

### Phenotypic characterization of LrS conditioned M2 macrophages

**Figure 4:**
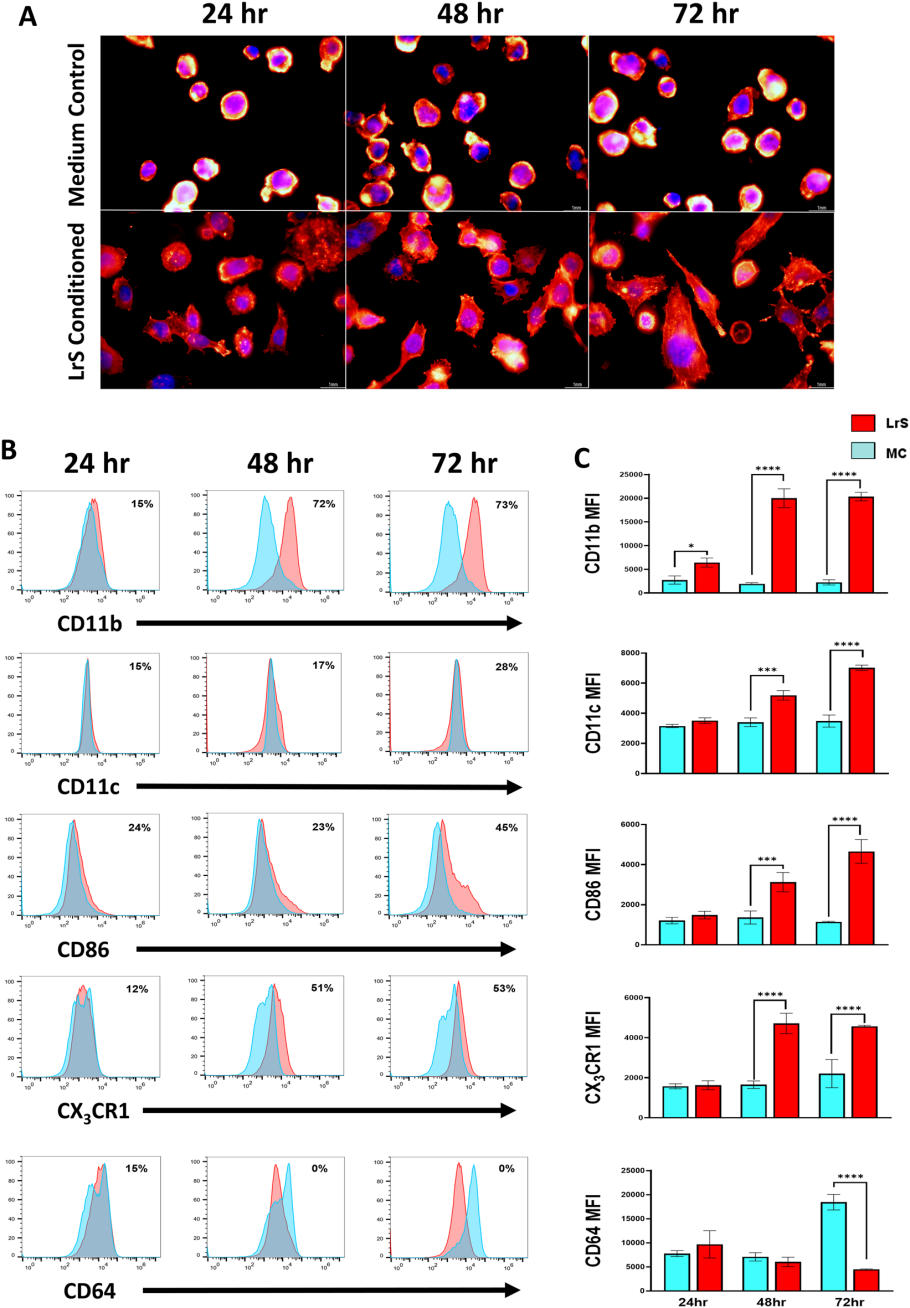
Conditioning with the LrS induces distinct morphological changes and increases the cell-surface expression of CD11b, CD11c, CX3CR1 and CD86 in THP-1 human monocytes. (**A**) Morphological confirmation of THP-1 monocyte differentiation into M2 macrophages following 72 h of conditioning with the LrS. Cells were stained with phalloidin-CF568 (red) and counterstained with DAPI (blue) (**B**) Histogram Overton subtraction was used to determine the relative number of cells expressing CD11b, CD11c, CD86, CX3CR1, or CD64 following conditioning with the LrS (red) over untreated (blue) controls. (**C**) Median fluorescence intensity (MFI) of THP-1 cells expressing CD11b, CD11c, CD86, CX3CR1, or CD64 was used to confirm the results of the histogram Overton subtraction. Data shown is the mean of the MFI ± SEM (n = 3). Significance is indicated as *p < 0.05, **p < 0.01, ***p < 0.001 as determined by one-way ANOVA and Tukey’s post-hoc test.

Phenotypic and morphological characterization of THP-1 human monocytes conditioned with the LrS was done to confirm the insights gained from the transcriptional and cytokine and chemokine profiling. Indeed, the LrS induced morphological changes in THP-1s over the three different time points examined, with the majority of cells appearing as differentiated macrophages following 72 h of conditioning (Fig. 4A). These morphological changes were in keeping with the transcriptional profiling, which indicated increased transcription of genes involved in myeloid leukocyte differentiation, cytoskeletal organization and adhesion (Figs. 1C, 2C). Flow cytometric analysis indicated a substantial shift in the number of cells expressing CD11b and CD11c, also indicative of monocyte differentiation into an antigen presenting cell (APC) phenotype (Fig. 4B). Probing these patterns further, histogram analysis revealed a 72% and 73% increase in the number of cells expressing CD11b following 48- and 72 h of LrS-conditioning (Fig. 4B). This correlated to a significant increase in CD11b median fluorescence intensity (MFI) when cells were conditioned with the LrS for 48- or 72 h, compared to medium controls (Fig. 4C). Numbers of CD11c^+^ cells were much lower than CD11b^+^ cells, even after 72 h of LrS conditioning (Fig. 4B). Cell surface expression of CD86, a co-stimulatory cell-surface molecule involved in T cell activation and survival, and of the fractalkine receptor CX_3_CR1 was also increased in cells conditioned with the LrS in a time-dependent fashion (Fig. 4B,C). Conversely, CD64 expression was significantly reduced in cells conditioned with the LrS following 72 h of conditioning (Fig. 4B,C).

**Figure 5:**
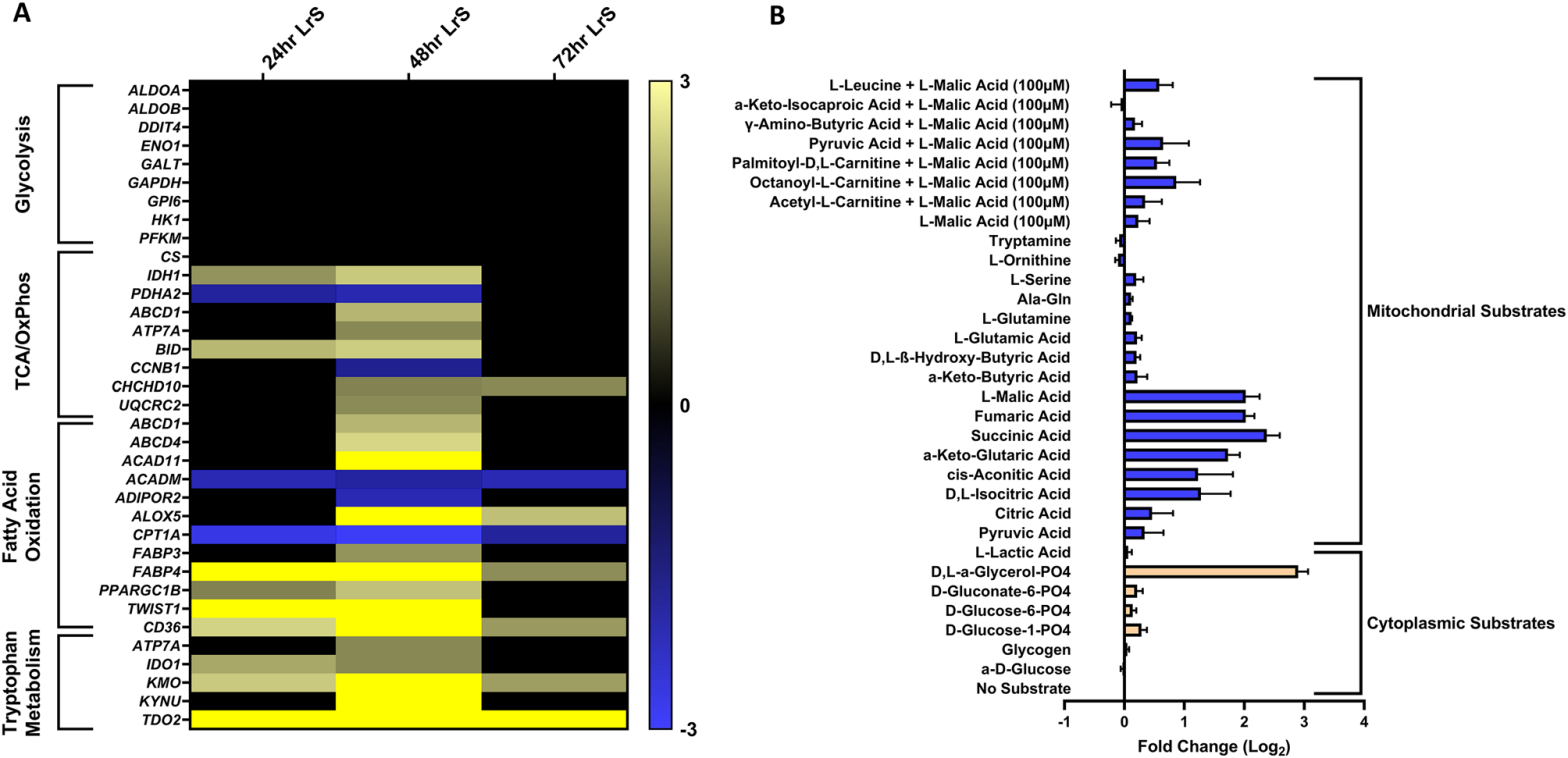
The LrS induces metabolic functional changes in THP-1 human monocytes. (**A**) Temporal changes in the transcription of genes involved in glycolysis, fatty acid oxidation, oxidative phosphorylation, and tryptophan metabolism. (**B**) Conditioning with the LrS results in different metabolic utilization signatures in THP-1 human monocytes. Data shown is the mean of the fold change (log2) ± SEM of substrate utilization when compared to an untreated control over 24 h (*n* = 3).

gProfiling analysis identified multiple different metabolic pathways influenced by LrS conditioning, and increased transcription of genes associated with immunoregulatory macrophage fatty acid and tryptophan metabolism was observed (Figs. 1, 2). As such, the impacts of the LrS on THP-1 metabolic signatures was investigated to ascertain whether the changes at the transcriptional, protein, and cell-surface molecule expression level also correlated to a metabolic signature consistent with immunoregulatory macrophage activity. Indeed, the LrS increased the transcription of many genes involved in fatty acid oxidation such as *ABCD1* (2.1 fold-change), *ABCD4* (2.5 fold-change), *TWIST1* (3.7 fold-change), *CD36* (8.34 fold-change), and *FABP4* (35 fold-change), and tryptophan metabolism such as *KMO* (11.9 fold-change) and *TDO2* (47.6 fold-change), with no changes in the transcription of genes involved in glycolysis following 48 h of conditioning (*n* = 4, *p* < 0.05) (Fig. 5A). Consistent with these results, metabolic utilization assays indicated that LrS conditioning did not increase the utilization rate of glycolytic intermediaries, suggesting that the levels of glycolytic flux were not impacted (Fig. 5B). However, LrS-conditioned macrophages had increased utilization of metabolites involved in the citric acid cycle, indicating an intact citric acid cycle (Fig. 5B).

### The LrS influences protein lysine acetylation patterns in THP-1 monocytes

**Figure 6:**
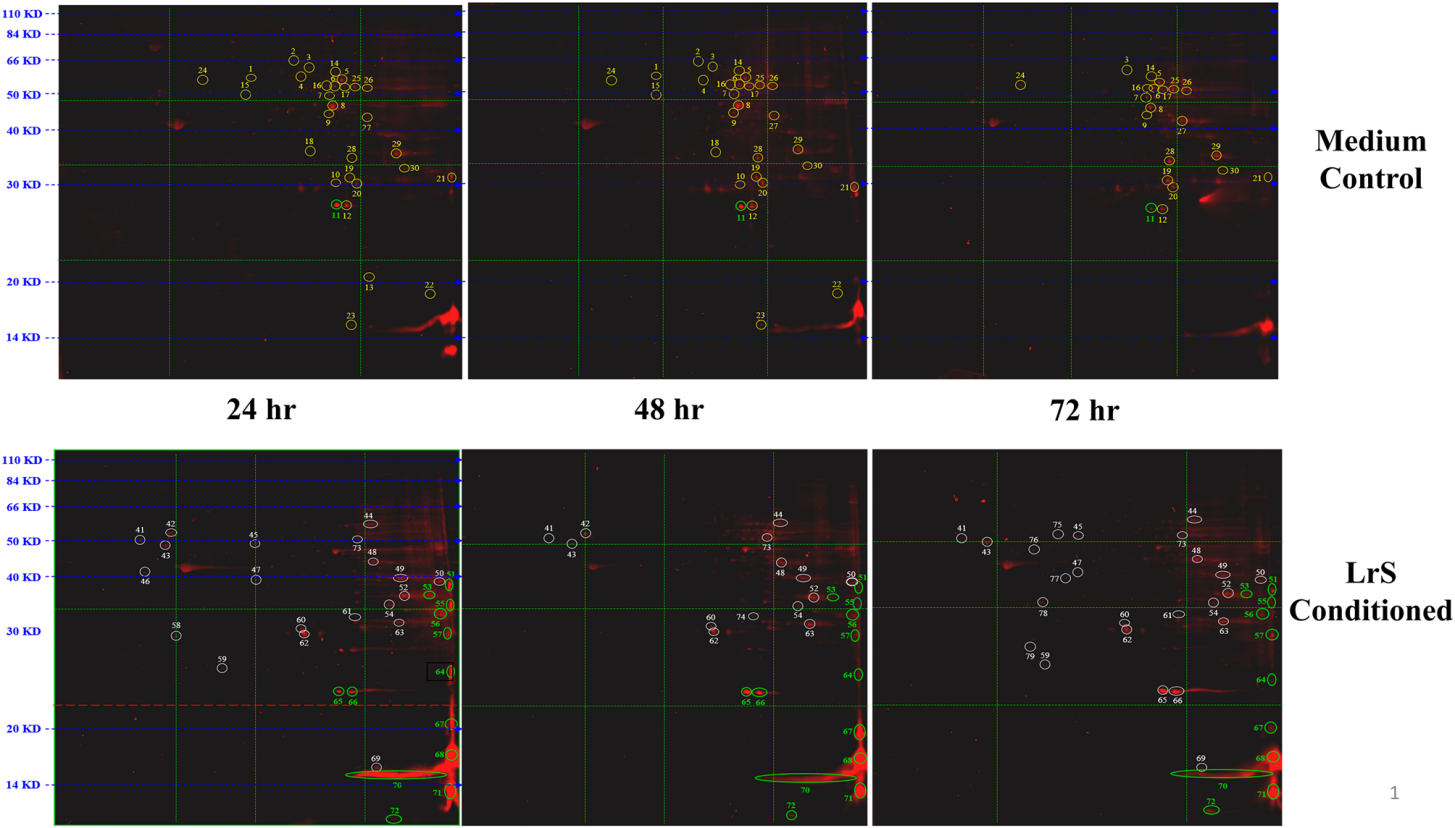
The LrS induces unique temporal changes in protein acetylation patterns in THP-1 human monocytes following conditioning for 24 h, 48 h, and 72 h. Two-dimensional western blots probed for anti-lysine acetylation identified unique protein acetylation patterns following LrS conditioning when compared to controls.

To further interrogate the mechanism(s) of action behind the observed phenotypic changes induced by conditioning with the LrS, post-translational changes via acetylation of protein lysine residues were examined. Two-dimensional western blot analysis determined that conditioning with the LrS resulted in post-translational modifications in 69 different proteins over the three different time points examined when compared to controls (Table 1; Fig. 6). Subsequent peptide mass fingerprinting revealed that conditioning with the LrS increased the acetylation of histones H3 and H4, confirming previously obtained results (Table 2-II). Moreover, the acetylation state of several proteins involved in macrophage mitochondrial and metabolic function were influenced by conditioning with the LrS. These included phosphoglycerate mutase 1, ATP synthase subunit O, mitochondrial superoxide dismutase, isocitrate dehydrogenase (NADP), and glyceraldehyde-3-phosphate dehydrogenase (Table 2).

**Table 1:** Fold change differences in the amount of acetylation of proteins detected in THP-1 human monocytes conditioned with the LrS versus medium controls for 24-, 48-, and 72-h.

Spot ID	24 h acetyl ratio (secretome/control)	48 h acetyl ratio (secretome/control)	72 h acetyl ratio (secretome/control)
1	1.3	− 1.3	0.0
2	− 4.3	2.5	0.0
3	1.0	− 1.2	1.6
4	− 1.3	− 1.6	0.0
5	− 1.5	− 1.9	− 2.0
6	− 3.6	− 2.9	− 4.5
7	− 2.9	1.2	− 2.0
8	− 1.2	− 1.7	1.3
9	− 1.5	1.0	1.1
10	− 1.6	− 2.2	0.0
11	− 8.3	− 7.9	− 1.8
12	− 1.6	− 3.3	− 3.0
13	− 10.4	0.0	0.0
14	− 1.5	− 2.0	− 3.3
15	1.0	− 2.2	0.0
16	− 3.6	− 1.6	− 2.2
17	− 1.5	− 3.4	− 3.0
18	1.4	1.4	0.0
19	1.6	− 2.3	− 3.8
20	1.5	− 2.5	− 1.8
21	− 2.8	− 4.2	1.5
22	− 3.7	− 1.0	0.0
23	2.4	− 2.1	0.0
24	1.5	− 2.0	− 5.8
25	− 1.3	− 1.4	− 3.1
26	− 1.7	− 1.2	− 1.9
27	2.2	− 1.8	− 3.8
28	2.2	− 1.6	− 2.5
29	− 3.4	1.4	− 4.2
30	2.4	1.6	− 1.2
41	3.0	− 1.1	− 1.0
42	3.7	17.8	0.0
43	2.2	1.7	1.3
44	4.7	2.3	4.5
45	5.5	0.0	4.0
46	2.7	0.0	0.0
47	1.6	0.0	1.2
48	6.3	1.8	1.1
49	2.4	2.3	1.0
50	2.6	3.5	1.3
51	19.8	4.5	5.3
52	1.3	2.6	2.9
54	2.8	3.2	1.2
55	15.2	1.5	6.1
56	26.2	2.1	10.0
57	2.0	4.9	6.7
58	7.2	0.0	0.0
59	2.2	0.0	3.6
60	2.9	1.5	1.3
61	2.0	0.0	1.7
62	3.1	1.5	4.6
63	2.6	3.2	− 1.1
64	30.7	5.2	19.5
65	37.0	308.5	113.3
66	53.4	39.1	50.1
67	37.1	20.0	3.3
68	7.0	1.7	3.7
69	3.5	1.1	3.1
70	11.0	6.0	4.0
71	5.6	35.1	43.4
72	10.6	12.3	7.2
73	2.0	2.4	3.1
74	0.0	3.0	0.0
75	0.0	0.0	7.2
76	0.0	0.0	1.6
77	0.0	0.0	3.8
78	0.0	0.0	1.6
79	0.0	0.0	2.7

**Table 2:** Protein identification of spot IDs showing a fold change ≥ 3 in acetylation of lysine residues in proteins found in THP-1 human monocytes conditioned with the LrS when compared to controls.

Spot #	Protein ID	Acetyl ratio 24 h	Acetyl ratio 48 h	Acetyl ratio 72 h
		Fold change secretome/control	Fold change secretome/control	Fold change secretome/control
11	Phosphoglycerate mutase 1	− 8.3	− 7.9	− 1.8
13	Cellular nucleic acid-binding protein	− 10.4	0.0	0.0
21	40S ribosomal protein	− 2.8	− 4.2	1.5
24	T-complex protein 1 subunit epsilon	1.5	− 2.0	− 5.8
29	Glyceraldehyde-3-phosphate dehydrogenase	− 3.4	1.4	− 4.2
42	Vimentin/Tubulin alpha-1C chain	3.7	17.8	0.0
44	Catalase	4.7	2.3	4.5
45	Heterogeneous nuclear ribonucleoprotein H	5.5	0.0	4.0
48	Isocitrate dehydrogenase [NADP], mitochondria	6.3	1.8	1.1
51	Heterogeneous nuclear ribonucleoproteins A2/B1	19.8	4.5	5.3
53	Heterogeneous nuclear ribonucleoproteins A2/B1	5.8	1.6	9.0
55	Prohibitin-2	15.2	1.5	6.1
56	Heterogeneous nuclear ribonucleoprotein A1	26.2	2.1	10.0
57	40S ribosomal protein S4, X isoform	2.0	4.9	6.7
58	Chloride intracellular channel protein 1	7.2	0.0	0.0
59	Proteasome subunit beta type-4/Actin Fragment	2.2	0.0	3.6
64	ATP synthase subunit O, mitochondrial	30.7	5.2	19.5
65	Superoxide dismutase [Mn], mitochondrial (SOD2)	37.0	308.5	113.3
66	Superoxide dismutase [Mn], mitochondrial (SOD2)	53.4	39.1	50.1
67	60S ribosomal protein L12	37.1	20.0	3.3
68	Histone H2B type 1-M	7.0	1.7	3.7
70	Histone H3.3	11.0	6.0	4.0
71	Histone H4	5.6	35.1	43.4
72	Profilin-1	10.6	12.3	7.2
75	Heterogeneous nuclear ribonucleoprotein H	0.0	0.0	7.2
77	Uroporphyrinogen decarboxylase/Cactin Fragment	0.0	0.0	3.8

### LrS conditioned THP-1 macrophages respond robustly to LPS challenge

**Figure 7:**
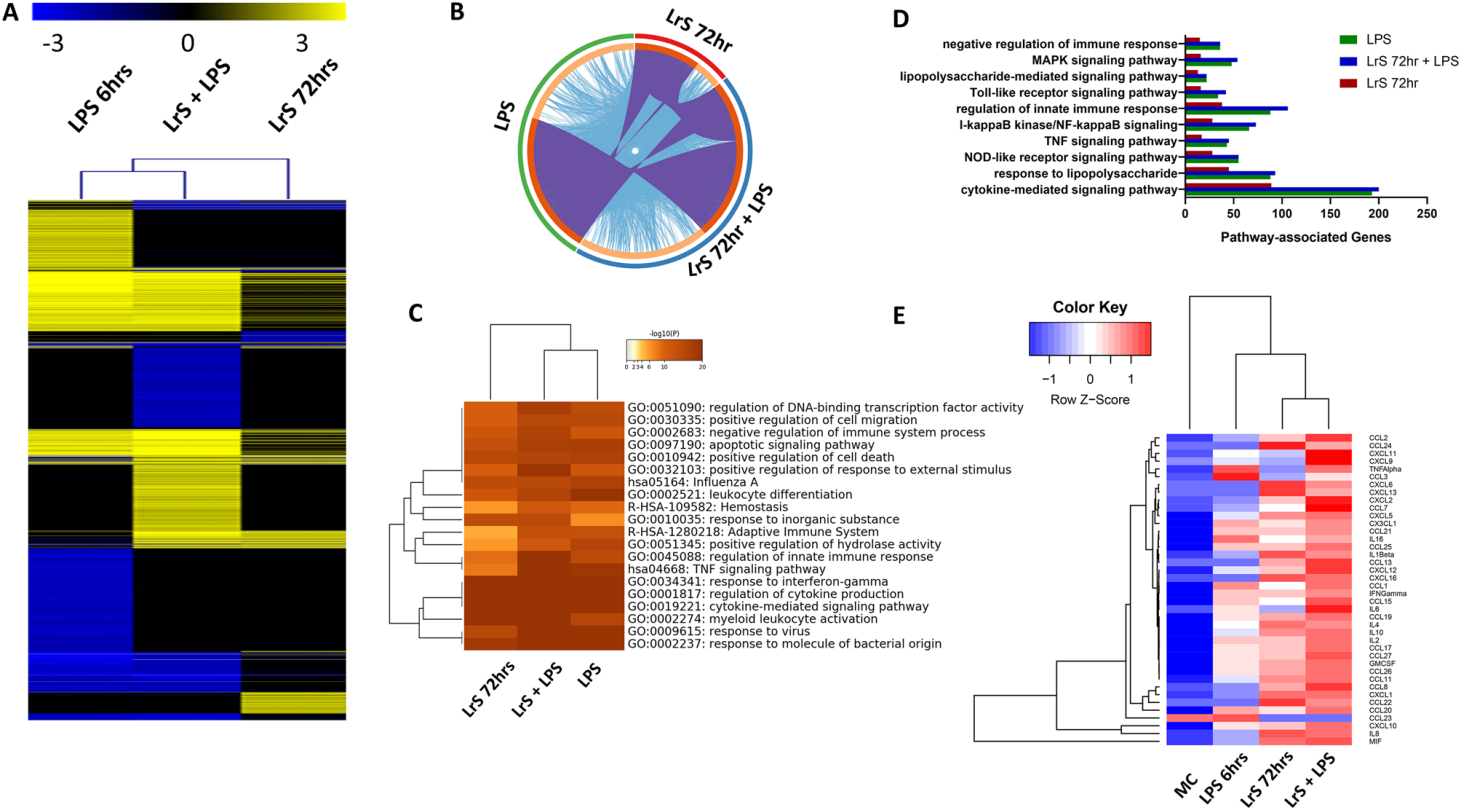
LPS-challenge of THP-1 monocytes conditioned with the LrS reveals heightened responsiveness. (**A**) Two-dimensional hierarchical clustering analysis of global gene expression patterns in THP-1 human monocytes challenged with LPS, conditioned with the LrS for 72 h, or conditioned with the LrS for 72 h and subsequently challenged with LPS for 6 h. (*n* = 4; *p* < 0.05; fold change different > 1.5 vs. untreated cells). (**B**) Circos plot visualization of gene overlap analysis and shared enriched ontologies for each of the challenges. Identical genes found in each gene set are coloured dark orange and are linked by purple lines while unique genes are coloured light orange. Blue lines link the different genes where they fall into the same ontology term. (**C**) Two-dimensional hierarchical clustering analysis of shared enriched ontology clusters between the different challenges. (**D**) Total number of genes which belong to functionally enriched immune-related pathways. (**E**) Two-dimensional hierarchical clustering analysis of cytokine and chemokine production profiles from THP-1 human monocytes in response to the different challenges. Data shown is the Z-score statistic for each cytokine and chemokine measured (*n* = 4).

LPS challenge of THP-1 monocytes was used to further examine overall immune effector function and cellular responses of macrophages to this key TLR4 ligand following conditioning with the LrS for 72 h. 2D hierarchical heat-map cluster analysis revealed that LrS conditioning of THP-1s prior to LPS challenge resulted in unique global gene expression profiles, distinct from LPS challenge or LrS conditioning alone (Fig. 7A). In fact, LrS-conditioned macrophages shared a very limited subset of differentially expressed genes and enriched pathway activation with LPS challenge alone (Fig. 7B,C). Moreover, the number of differentially expressed genes within key immune-related signaling pathways in macrophages was significantly lower in THP-1 monocytes conditioned with the LrS compared to LPS challenge alone (Fig. 7D), indicating that the unique transcriptional signature in response to LrS conditioning in THP-1 macrophages is distinct from the signature induced by LPS challenge. LPS challenge of LrS-conditioned macrophages resulted in increased expression of genes in the Toll-like receptor signaling, NF-κB signaling, regulation of the innate immune response, and cytokine-mediated pathways when compared to LPS or LrS conditioning alone (Fig. 7D). Cytokine and chemokine profiling confirmed these results, as LrS conditioning enhanced production of LPS-induced cytokine and chemokine production (Fig. 7E; Figs. S3 and S4) coupled with increased production of immunoregulatory IL-10 and IL-1Ra, suggesting that conditioning of THP-1 monocytes with the LrS influences subsequent immune outcomes to LPS challenge.

### LrS maintains its immunoregulatory activity in combination with RA in PMA-differentiated THP-1 cells

**Figure 8:**
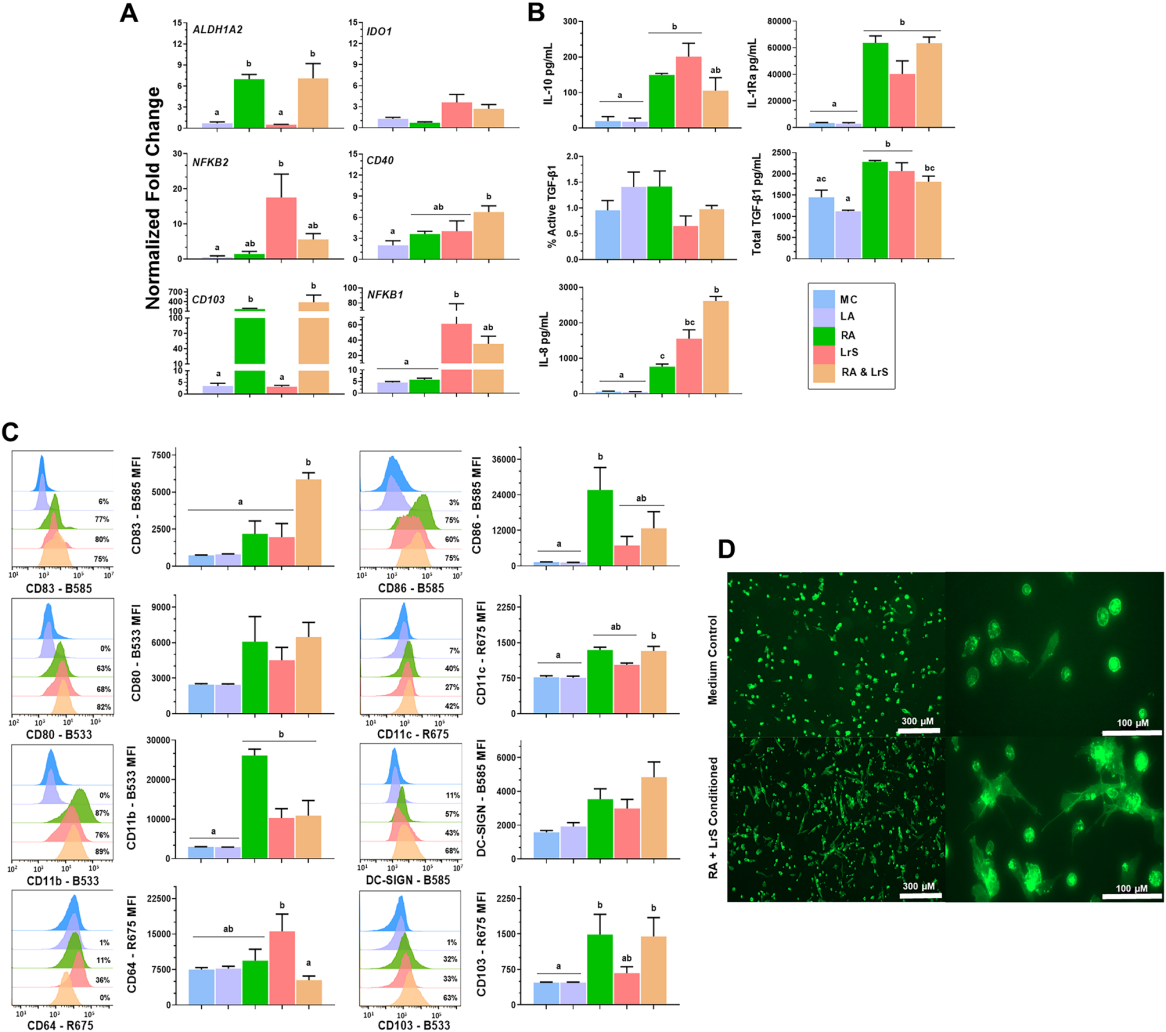
LrS and RA co-conditioning imprints an immunoregulatory profile in PD-THP-1 macrophages. Different letters denote significant differences (*p* < 0.05) as determined by one-way ANOVA and Tukey’s post-hoc test. (**A**) Gene expression in PD-THP-1 macrophages conditioned with l-lactic acid, RA, LrS and LrS + RA. The mean fold change expression (mean ± SEM (*n* = 3)) was normalized to baseline expression in PD-THP-1 macrophages. (**B**) Cytokine production in PD-THP-1 macrophages conditioned with l-lactic acid, RA, LrS and LrS + RA. Bar graphs represent mean cytokine production (mean (pg/mL) ± SEM (*n* = 3)) for IL-10, IL-1Ra, % active TGF-β1, total TGF-β1 and IL-8. (**C**) Expression of cell surface markers on PD-THP-1 macrophages conditioned with l-lactic acid, RA, LrS and LrS + RA. The histograms and Overton subtraction values were generated using FlowJo software. Overton subtraction values represent the percentage of cells expressing CD83, CD80, CD86, CD64, CD103, CD209, CD11b and CD11c. Histograms represent one of three biological replicates. Bar graphs represent mean MFI values (mean MFI ± SEM (*n* = 3)). (**D**) Morphological changes in PD-THP-1 macrophages induced by RA and LrS co-conditioning over 48 h; PD-THP-1 macrophages were then stained with phalloidin-CF488.

Cell surface marker profiles, gene expression and cytokine production were measured following LrS + RA conditioning of PD-THP-1 cells to investigate effects on differentiated macrophages and potential interactions of the LrS with RA, a key mucosal immunoregulatory mediator. Co-conditioning of PD-THP-1 cells with LrS + RA resulted in differential *ALDH1A2* expression at 24 h (*n* = 3, *p* < 0.001) (Fig. 8A), returning to PD-THP-1 baseline levels at 48- and 72 h. RA, LrS or co-conditioning did not significantly affect expression of *IDO1,* a key immunoregulatory gene. *NFKB1* expression was significantly increased at 48 h in LrS-conditioned PD-THP-1 cells (*n* = 3, *p* < 0.05) and declined after 72 h (Fig. 8A), and this effect of the LrS was not significantly reduced by co-conditioning with RA. In keeping with the increased CD103 cell surface marker expression, *CD103* was also upregulated by RA and by LrS + RA co-conditioning (*n* = 3, *p* < 0.001) (Fig. 8A). LrS conditioning significantly increased *NFKB2* expression, while *CD40* expression was increased by LrS + RA co-conditioning (Fig. 8A). Conditioning of PD-THP-1 cells with the LrS alone or in combination with RA increased production of immunoregulatory mediators IL-10 (*n* = 3, *p* < 0.05) and IL-1Ra (*n* = 3, *p* < 0.001), and maintained constitutive production of TGF-β1 relative to LA controls (Fig. 8B). LrS + RA co-conditioning also enhanced production of chemokine IL-8, potentially reflecting the LrS-induced increase in expression of *NFKB1* and *NFKB2* at 48 and 24 h respectively. Overall, co-conditioning with LrS + RA differentiated THP-1 macrophages into a mature APC phenotype with characteristics of immunoregulatory intestinal monocyte-derived dendritic cells.

Co-conditioning of PD-THP-1 cells with LrS + RA increased the percentage of cells expressing CD83 over all three time points, with conditioning for 72 h inducing the highest number of CD83^+^ cells (*n* = 3, *p* < 0.001) relative to baseline PD-THP-1 CD83 expression (Fig. 8C). Co-conditioning of PD-THP-1 macrophages with LrS + RA also significantly increased the number of CD103^+^ cells after 72 h (*n* = 3, *p* < 0.05) (Fig. 8C), as well as the number of cells expressing the co-stimulatory molecules CD80 and CD86. In contrast, cell surface expression of the proinflammatory marker CD64 was reduced by LrS + RA co-conditioning relative to conditioning with LrS alone. Co-conditioning also increased numbers of CD11b^+^ cells (*n* = 3, *p* < 0.001), CD11c^+^ cells (*n* = 3, *p* < 0.001) and CD209^+^ cells (*n* = 3, *p* < 0.05). PMA differentiation of monocytic THP-1 cells generally results in morphological changes; PD-THP-1 co-conditioned with LrS + RA had a distinctive appearance, with elongated extensions typical of differentiated APC morphology (Fig. 8D).

## Discussion

Regulation of macrophage activity at the gut-mucosal interface is integral for maintaining immune homeostasis and shaping host immune outcomes to subsequent pro-inflammatory challenge. Microbiota-induced innate immune memory has been described as a potential mechanism through which the resident microbiota induces immune tolerance, with some studies suggesting that these signals delivered through PRRs can also induce innate immune training to subsequent inflammatory signals^25–28^. Recent evidence suggests that these varied modes of communication may be an important route through which LAB and other members of the gut microbiota exert physiological and immunological changes at distal and systemic sites within the host^29–31^.

In the present study, we determined that the LrS induced a unique transcriptional profile in THP-1 monocytes depending on exposure time, with a high degree of differential gene transcription occurring after 48 h and a substantial reduction in gene expression following 72 h of LrS conditioning. This is, to our knowledge, the first report of temporal macrophage transcriptional reprogramming by prolonged exposure to a LAB-derived secretome. Transcriptional regulation of macrophage polarization is well documented, with many gene expression profiles recognized as associated with monocyte differentiation and immunoregulatory macrophage activation^4,32–34^. The LrS induced increased transcription of *ADAMDEC1*, *ZFP36L1*, and *RND3*, genes and transcription factors involved in monocyte differentiation into mature macrophages^35–37^ and gene enrichment analysis identified differential activation of cellular pathways involved in cytoskeletal rearrangement and myeloid leukocyte differentiation. Our previous analysis has indicated that the LrS may influence histone acetylation patterns within challenged intestinal epithelial cells by decreasing global histone acetylation and thereby attenuating pro-inflammatory gene transcription. Here we show that the LrS may increase H3 and H4 histone acetylation in a temporal fashion in THP-1 human monocytes. This may help to explain the observed increase in overall differential gene expression and subsequent changes in protein expression patterns and cell morphology. We also examined whether the LrS could influence DNA methylation patterns in these same cells. Unlike histone acetylation patterns, the LrS did not have an impact on global DNA methylation.

Morphological and flow cytometric analysis confirmed these findings, as LrS conditioning of THP-1 monocytes resulted in temporal morphological changes, in keeping with differentiation to a more mature myeloid phenotype, with dendrites to increase cell surface area and facilitate monitoring of the gut environment^20,38^. Lrs conditioning also induced an increase in the cell surface expression of CD11b and CD11c, indicative of monocyte to macrophage differentiation, and increased expression of the fractalkine receptor CX_3_CR1. CX_3_CR1-expressing macrophages in the GALT have been shown to play an integral role in maintaining gut homeostasis by producing IL-10 and are involved in facilitating cross-talk between the resident gut microbiota and the underlying immune cell population^39–45^. Conversely, LrS-conditioned THP-1s had reduced cell-surface expression of CD64. Human peripheral blood mononuclear cell-derived macrophages treated with LPS or IFN-γ express high levels of membrane-bound CD64 and display a phenotype consistent with M1-macrophage activity^46,47^. Moreover, administration of an immunotoxin that targets CD64 led to the selective elimination of M1 macrophages in an inflammatory disease setting, illustrating the negative outcomes of dysregulated activity of M1 phenotype macrophages and highlighting the importance of CD64 in M1-associated macrophage activity^48,49^.

Changes in expression of multiple immunoregulatory macrophage-associated genes was also observed following conditioning with the LrS, suggesting polarization of THP-1 monocytes into immunoregulatory macrophages. For example, the LrS induced the expression of *ATF3* and *DUSP1*, key regulators of innate immune activity, which is consistent with our previous results examining the LrS impact on TNFα and Salmonella secretome-challenged HT-29 IEC^13^. Overexpression of ATF3 has been shown to inhibit inflammatory macrophage gene transcription and activate expression of immunoregulatory macrophage genes through the Wnt/β‑catenin signaling pathway^50^. Further, DUSP1 polarizes macrophages towards an M2 phenotype via inhibition of the AP-1 transcription factor complex, inhibiting inflammatory gene transcription^32^.

Cytokine and chemokine profiling confirmed these findings, as we observed increased production of immunoregulatory macrophage-associated cytokines such as IL-10 and IL-1Ra following conditioning with the LrS. IL-10 production is associated with immunoregulatory M2 macrophage activity and polarization as it serves multiple roles in the regulation of inflammatory immune responses^51^. It binds to the IL-10 receptor (IL-10R), resulting in the phosphorylation of STAT3 and subsequent transcription of genes involved in immunoregulation and repression of pro-inflammatory cytokine production^52^. Indeed, IL-10 signaling was identified by GSEA analysis as being impacted by the LrS and *IL-10R* expression was increased following LrS conditioning. These cellular insights into LrS-mediated macrophage polarization into an immunoregulatory phenotype implicate enhanced IL-10 signaling in THP-1 monocytes. IL-1Ra antagonizes the activity of IL-1α and IL-1β by binding to the IL-1R1 receptor and is thought to play an integral role in the resolution of the inflammatory response as it is typically produced following IL-1 secretion. We also observed increased transcription of *IL1R2* (Interleukin 1 Receptor Type 2), a decoy receptor for IL-1β/α and a known marker of immunoregulatory M2 macrophages^53^, following 24- and 48 h of conditioning, suggesting that the LrS significantly alters pro-inflammatory IL-1 signaling outcomes. Additionally, LrS-conditioned THP-1 monocytes produced IL-4, an immunoregulatory and pleiotropic cytokine which inhibits the production and secretion of pro-inflammatory cytokines and can promote and shape adaptive immune responses^54^. As was seen with IL-10/*IL-10R* production and expression, the LrS also induced the expression of the IL-4 receptor, *IL4Rα*, as well as *IL4I1* (IL-4-induced gene 1), an L-amino acid oxidase and M2 immunoregulatory macrophage associated marker with inhibitory roles in T cell activation^55^. IL-4 signaling in macrophages promotes IL-1Ra production and IL1R2 expression^56^. We also observed increased production of Type I interferons from LrS-conditioned monocytes, suggesting enhanced anti-viral capacity. Evidence for microbiota-induced Type I interferon production in APCs suggests that this is an important route for instructive transcriptional and metabolic programming, enabling robust responses to subsequent pathogen challenge by promptly activating the adaptive immune response^57^.

Macrophage immunometabolism plays an integral role in macrophage effector function. M1 macrophages are characterized by their increased reliance on aerobic glycolysis for energy, resulting in a substantially higher glycolytic flux and a shunted citric acid cycle in order to maintain sustained cytokine and inflammatory mediator production. In contrast, M2 macrophages rely on oxidative phosphorylation mediated through an intact citric acid cycle, highlighting key metabolic differences which can be used to distinguish macrophage phenotype and effector functions^58,59^. For this reason, we examined the ability of the LrS to influence metabolic flux in THP-1 monocytes. We observed increased utilization of all citric acid cycle intermediates, without altered glycolytic metabolism, indicative of an immunoregulatory macrophage metabolic signature. Moreover, the LrS increased transcription of *CD36*, a cell-surface receptor involved in fatty acid uptake, and *FABP4* (Fatty Acid Binding Protein-4), a cytoplasmic protein involved in lipid transport and metabolism^60^. Lipid metabolism was identified by gProfiler as one of the metabolic pathways influenced by the LrS and is a hallmark of immunoregulatory macrophage activity. *TDO2* (Tryptophan 2,3-Dioxygenase), a key enzyme involved in tryptophan metabolism and activation of the kynurenine pathway which plays an integral role in M2 macrophage activity^61^, was also up-regulated in LrS-conditioned THP-1 monocytes, further suggesting that the LrS influences multiple macrophage metabolic pathways.

To determine potential underlying mechanism(s) of action behind the observed changes in metabolic activity and functional outcomes in LrS-conditioned THP-1 monocytes, post-translational modifications of intracellular proteins were examined. This analysis revealed the deacetylation of phosphoglycerate mutase 1 (PGAM1) in THP-1s conditioned with the LrS. PGAM1 catalyzes a rate-limiting step of glycolysis in leukocytes by converting 3-phosphoglycerate (3-PG) to 2-phosphoglycerate (2-PG). When deacetylated, PGAM1 exhibits diminished activity^62^, suggesting a possible mechanism of action through which the LrS alters metabolism in THP-1s by reducing glycolytic activity within the cell. Moreover, LrS conditioning led to deacetylation of GAPDH, another key enzyme involved in glycolysis. Deacetylation of cytosolic GAPDH results in decreased glycolytic activity in response to glucose^63^, providing additional mechanistic evidence for reduced glycolytic flux in THP-1 monocytes conditioned with the LrS. In contrast, mitochondrial isocitrate dehydrogenase 2 (IDH2), a key enzyme involved in the oxidative decarboxylation of isocitrate to α-ketoglutarate, was acetylated following LrS-conditioning. Unlike isocitrate dehydrogenase 3 (IDH3), the reaction catalyzed by IDH2 is reversible and uses NADP + and NADPH instead of NAD + and NADH as a redox couple. This leads to the accumulation of NADPH in the mitochondria, which can be used for scavenging mitochondrial ROS (mtROS)^64^. Moreover, IDH2 has also been implicated in the export of citrate from the mitochondria for lipid biosynthesis, a hallmark of M1 macrophage activity, following reductive carboxylation^65^. When IDH2 becomes acetylated, it has reduced activity^66^, which may lead to increased flux of isocitrate through IDH3 and a potential reduction in mtROS scavenging due to impaired NADPH generation. However, mitochondrial manganese superoxide dismutase (SOD2), an enzyme which typically displays dismutase activity by converting superoxide to O_2_ or H_2_O_2_, was acetylated following conditioning with the LrS. Acetylated SOD2 displays peroxidase activity yet has increased capacity for the rapid generation of mitochondrial H_2_O_2_ through a mechanism which is still not fully understood^67,68^. Increased levels of mitochondrial H_2_O_2_ within macrophages have been shown to drive M2 polarization^69^, potentially through the recruitment and stabilization of hypoxia-inducible factor-2α (HIF-2α) which can act to suppress nitric oxide synthesis^70^. Although other proteins were identified with differential lysine acetylation patterns following conditioning with the LrS, more research is needed to ascertain the diverse functional outcomes of these post-translational modifications, especially in the context of host-microbe interactions. Indeed, post-translational modification of proteins involved in metabolism has emerged as a potential route through which metabolic activity is regulated^71^, and the results presented here may represent a novel means of host-microbe communication through which soluble components derived from LAB can regulate the activity of many important cellular processes.

The ability of macrophages to respond robustly to pathogen-associated molecular patterns is integral to host defense and survival. LPS challenge of LrS-conditioned THP-1 monocytes revealed heightened responsiveness, suggesting a degree of innate immune priming, while maintaining an overall M2 phenotype. Evidence for alterations of macrophage responses to pro-inflammatory challenge indicate strain-specific impacts on immune outcomes as some strains within the same species have antagonistic impacts on PRR-induced cytokine release^72^. For example, *Lacticaseibacillus paracasei* attenuates LPS-induced cytokine production by PMA-differentiated THP-1 cells^73^ while exopolysaccharide isolated from *L. paracasei* DG stimulates cytokine production from THP-1 monocytes^74^. PRR-mediated recognition of microbiota-derived peptidoglycan by neutrophils has been shown to enhance and prime systemic immunity towards *Streptococcus pneumoniae* and* Staphylococcus aureus* infection^26^, suggesting that this priming of the innate immune system by LAB and other members of the microbiota can influence systemic immune responses and promote effective host defence against pathogens.

RA is a key mediator involved in generating tolerogenic APC phenotypes in the gut mucosal environment. To explore potential interactions between RA and LrS on differentiated macrophages, effects were investigated by measuring gene expression, cell surface marker and cytokine profiles of PD-THP-1 cells that are characteristic of either tolerogenic or proinflammatory APC phenotypes. In keeping with the effects of LrS on THP-1 monocytes and with our previous work examining the impact of milk fermented with *L. rhamnosus* R0011 on macrophages, LrS + RA co-conditioning of PD-THP-1 macrophages conferred an overall immunoregulatory phenotype. Similarly to LrS conditioned THP-1 monocytes, we observed an increase in the number of CD11b^+^ and CD11c^+^ cells and a reduction in CD64^+^ cell numbers in co-conditioned PD-THP-1 cells. We also observed an increased number of cells expressing CD103, costimulatory molecules CD80 and CD86, and of CD209, an M2 macrophage phenotype marker^75^, following co-conditioning with LrS + RA. Co-conditioning also increased the number of cells expressing CD83, an important cell surface molecule involved in facilitating APC-T cell interactions^70,76,77^. In accordance with the increased number of CD103^+^ cells, *CD103* and *ALDH1A2* expression was induced by LrS + RA conditioning. Retinal dehydrogenase converts retinal to RA, and RA itself can act as a transcriptional factor inducing the expression of *ALDH1A2* in a positive feedback manner^78^. CD103^+^ APCs in the intestinal mucosa acquire a tolerogenic phenotype through an RA-dependent mechanism, driving the differentiation of gut homing regulatory T cells and maintaining mucosal tolerance^79,80^. Although we did not observe antagonistic nor synergistic interactions between LrS and RA, co-conditioning of the PD-THP-1 macrophages with LrS + RA increased the production of key regulatory cytokines IL-10 and IL-1Ra, as was observed for LrS-conditioned THP-1 monocytes. Interestingly, a *Limosilactobacillus reuteri* secretome has also been shown to influence the recall responses of human peripheral blood monocyte-derived dendritic cells and their RA-differentiated gut DC phenotype^28^, further illustrating the potential of secretome-mediated immunomodulatory activity of lactic acid bacteria.

Taken together, the results presented here describe transcriptional and functional re-programming of THP-1 human monocytes mediated by postbiotic secretome components of *L. rhamnosus* R0011. These secretome-conditioned macrophages showed functional, transcriptional, and immunometabolic signatures consistent with M2 immunoregulatory activity, with increased production of immunoregulatory cytokines IL-10, IL-1Ra, and IL-4 and a cell-surface expression profile of CD11b, CD11c^lo^, and CX_3_CR_1_, features shared with subsets of gut macrophages^81,82^. Moreover, LrS-conditioning of THP-1 monocytes did not impair responses to subsequent LPS challenge, despite their overall homeostatic M2 phenotype. Gut-associated macrophages play a dynamic and multifaceted role in host-microbe interactions and are required to respond appropriately to diverse gut microbe-derived cues to shape subsequent immune effector signals. The results reported here provide insight into novel routes for indirect secretome-mediated microbe-host communication and the impact on activation of multiple immune mechanisms integral for macrophage function and activity. To our knowledge, this is the first report of immunometabolic reprogramming of THP-1 human monocytes by LAB-secretome components. While outside the scope of our study, proteomic and metabolomic profiling of the LrS and of other lactobacilli secretomes will be important avenue to deepen understanding of these types of microbe-host interactions and gain insight into potential postbiotic mechanisms. Future work to examine the role of secretome-mediated communication in the impact of LAB and other gut microbes in host defense at the gut mucosal interface and distal systemic immunological outcomes in vivo may aid in elucidating gut microbe-mediated effects on systemic immunity.

## Experimental procedures

### Bacterial culture

Lyophilized *Lacticaseibacillus rhamnosus* R0011 was obtained from the Rosell Institute for Microbiome and Probiotics (Montreal, QC). The LrS was prepared as previously described^13,83^. Briefly, *Lacticaseibacillus rhamnosus* R0011 was grown in de Mann, Rogosa, and Sharpe broth (Oxoid, cat. # CM0359) overnight to stationary growth phase. Resulting cultures were further propagated in non-supplemented Roswell Park Memorial Institute (RPMI)-1640 (Sigma-Aldrich, cat. # R6504) to stationary phase, centrifuged at 3 000×*g* and filtered through a 0.22 µm filter to remove the bacteria. The filtrate (LrS) was immediately frozen at − 80 °C. A medium control consisting of only MRS diluted in RPMI-1640 medium was included and subjected to the same culture conditions and filtration process. The pH of the LrS was measured and the pH of the medium control was adjusted to that of the bacterial culture using l-lactic acid and concentrated HCl. The Megazyme d-/l-lactic acid kit was used to determine the l-lactic acid concentration in the LrS secretome, and equal amounts of l-lactic acid were added to the medium control to account for this potentially bioactive metabolite.

### THP-1 human monocyte conditioning and LPS challenge

The THP-1 human peripheral blood monocyte cell line (ATCC # TIB-202) was maintained in RPMI-1640 medium supplemented with 0.05 mM β-mercaptoethanol, 10% calf serum and 0.05 mg/mL gentamicin in a humidified incubator at 37 °C and 5% CO_2_. THP-1 monocytes were enumerated, and viability determined using Trypan Blue following sub-culturing. THP-1 monocytes were seeded into T25 tissue culture flasks or 96-well tissue culture plates at a concentration of 1 × 10^6^ cells/mL and used at a final concentration of 5 × 10^5^ cells/mL in non-supplemented RPMI-1640 medium. To determine the temporal effects of the LrS on monocyte differentiation, the LrS was added at a final concentration of 20% v/v to the THP-1 cells for 24-, 48-, and 72-h of conditioning, time points based on our prior work with the LrS^13,19,83^ and on standard differentiation protocols for these cells^21,22^.

For LPS challenges, THP-1 cells were cultured with lipopolysaccharide (LPS) (100 ng/mL) for 6 h alone or following conditioning with the LrS for 72 h. To characterize LrS impacts on differentiated macrophages, THP-1 monocytes were treated with 100 ng/mL phorbol 12-myristate 13-acetate (PMA) for 48 h in serum free media, then rested for 24 h in complete media^21^. PMA-differentiated cells (PD-THP-1) were also conditioned with a combination of RA (300.4 ng/mL), GM-CSF (100 ng/mL) and IL-4 (50 ng/mL) for 24-, 48- and 72-h (with or without LrS), in order to induce further differentiation to an intestinal dendritic cell-like phenotype and to assess LrS-mediated effects in concert with RA^28,84^.

Total RNA was harvested using the phenol-based TRIzol method of RNA extraction following manufacturer’s protocols (ThermoFisher Scientific, MA, USA). Briefly, 2 mL of TRIzol reagent was added to each culture flask to lyse the THP-1 cells. Cell culture homogenates were added to Phase Lock Gel-Heavy tubes for phase separation of total RNA. Total extracted RNA was then purified using the RNeasy Plus Mini Kit (Qiagen, Hildon, Germany). The purity and quality of RNA was determined using both the ND100 NanoDrop and an Agilent 2000 Bioanalyzer, respectively. Only samples with an RNA Integrity Number (RIN) greater than 9.0 were used for microarray analysis.

### Reverse transcription (RT) of RNA and direct-method of labelling

Control and experimental total RNA (15 µg) was reverse transcribed with Superscript IV (Invitrogen, MA, USA) and labelled with Cy3-dCTP and Cy5-dCTP (GE Healthcare, Amersham Biosciences) using the direct method of dye labelling as previously described^11,85^. Briefly, 3 µg/µL of oligo dT23 primers were added to the RNA and samples were heated to 70 °C for 30 min in order to reduce secondary structure formation. A cDNA synthesis master mix containing 5× First Strand Buffer, 0.1 M DTT, dNTPs, 200U of SuperScript IV, and either 1 mM of Cy3 or Cy5 dye were added and the samples were heated at 42 °C for 3 h in order to allow the RT to occur. Dye swaps between treated and control RNA were done to eliminate bias of dye labelling. Purification of the cDNA product was done using the QIAQuick PCR purification kit following manufacturer’s protocols (Qiagen). Labelling efficiency was determined by calculating the dye incorporation rate to ensure consistency between experiments.

### Microarray analysis

Hybridization of the labelled cDNA to the microarray was done using previously established protocols^11,12^. Following hybridization, the microarrays were scanned using the ScanArray 5000 instrument from Perkin-Elmer (Waltham, MA, USA) and spot intensities were quantified using ImaGene® version 9.0 (BioDiscovery, CA, USA). Normalization was done using locally weighted scatterplot smoothing (LOWESS)^85^. Statistical analyses and two-dimensional hierarchical clustering analyses was performed with Multi-Experiment Viewer (MeV, version 4.2). Genes with statistically significant changes in expression levels were selected based on a t-test yielding a *p*-value < 0.05 and a 1.5-fold-change gene expression cut-off. gProfiler, Gene Set Enrichment Analysis (GSEA) and EnrichmentMap pathway enrichment analyses were used in order to ascertain the pathways in which genes were significantly modified by the different treatments^86,87^. For some challenges, Metascape was used for circos plot generation and functional gene enrichment analyses^88^.

### Comparative RT-qPCR

Following conditioning of PD-THP-1 cells with RA and/or LrS, total RNA was harvested using the phenol-based TRIzol method of RNA extraction (Thermo Fisher Scientific) as described above. RNA was isolated and purified from three separate independent experiments for each treatment. Then, the total RNA was reverse transcribed with Superscript IV following manufacturer’s protocols and as previously described^13^. Briefly, oligoDT primers (50 µM), dNTPs (10 mM) and RNase free water was added to the highly purified RNA samples and heated to 65 °C for 5 min. A cDNA master mix containing 5X first strand buffer, DTT (0.1 M), and Superscript IV was added to the reaction mixture and incubated at 50 °C for 50 min to allow cDNA synthesis to occur. The reaction was heated to 70 °C for 15 min to inactivate the enzyme. Reverse-transcribed cDNA was diluted 1:5 in RNase-free water and stored at − 20 °C for RT-PCR. To determine transcript abundance of differentially expressed genes, RT-qPCR was done per the MIQE guidelines^89^. For the RT-qPCR reactions, 2.5 µl of diluted cDNA was used with gene-specific primers (Table 3) and SsoAdvanced Universal SYBR Green Supermix (Bio-Rad, CA, USA) per the manufacturer’s instructions. A total of 40 cycles consisting of template denaturation (15 s at 95 °C) and one-step annealing and elongation (30 s at 60 °C), with a Bio-Rad CFX Connect instrument (Bio-Rad, CA, USA) was used. The fold change expression levels were normalized to the expression levels of the media control and reference genes (*RPLPO* & *B2M*) using Bio-Rad CFX Manager 3.1. Statistical analysis was done using SigmaPlot (Version 14.5) ANOVA and further analysis with Tukey’s multiple comparison test when ANOVA indicated significant differences were present. All data are shown in bar graphs were generated using GraphPad Prism (Version 9) as mean normalized fold expression ± SEM to compare transcriptional abundance of specific gene.

**Table 3:** Primers used for RT-PCR to determine gene expression in conditioned PD-THP-1 cells.

Gene	GenBank accession number	Primer sequence (5′–3′)
*ALDH1A2*	NM_003888.4	F: GATAGAGATGCCCGGCGAG
		R: GACGTCCCCTTTCTGAAGCA
*CD40*	NM_001250.6	F: GGCAGGCACAAACAAGACTG
		R: TGGCTTCTTGGCCACCTTTT
*CD103*	NM_002208	F: AGGTCATCTGCTCATGTTTCAGT
		R: GCTCCAAAGAGGTTCTCCCC
*IDO1*	NM_002164.6	F: GCCTGATCTCATAGAGTCTGGC
		R: TGCATCCCAGAACTAGACGTGC
*NFKB1*	NM_003998.3	F: GCAGCACTACTTCTTGACCACC
		R:TCTGCTCCTGAGCATTGACGTC
*NFKB2*	NM_001261403	F:CAGACGAGTGTGGTGAGCTT
		R: GCTTGTCTCGGGTTTCTGGA
*B2M*	NM_004048	F: GTGCTCGCGCTACTCTCTC
		R: GTCAACTTCAATGTCGGAT
*RPLPO*	NM_001002	F: GCAATGTTGCCAGTGTCTG
		R: GCCTTGACCTTTTCAGCAA

### Global H3/H4 histone acetylation and DNA methylation determination

Global H3 and H4 histone acetylation patterns in THP-1 monocytes following conditioning with the LrS was determined as previously described^13^ the EpiQuik Global Histone H4 (P-4009) and H3 (P-4008) Acetylation Kits following manufacturer’s protocols (EpiGentek). Global DNA methylation patterns were determined using the MethylFlash Methylated DNA Quantification kit (P-1034) following manufacturer’s protocols (EpiGentek). Briefly, total DNA was extracted from THP-1 monocytes conditioned with the LrS or culture medium controls over 24-, 48-, or 72-h, using the PureLink Genomic DNA Mini Kit (Invitrogen). A total of 100 ng of DNA was spotted onto strip wells and assayed for methylated DNA. A positive control consisting of a known amount of methylated DNA was included, and the percentage of methylated DNA was calculated by comparing treated and untreated controls. Statistical analysis was done using GraphPad Prism one-way ANOVA and Tukey’s multiple comparison test when the ANOVA indicated significant differences were present. All data are shown as the mean percentage of change in histone acetylation or methylated DNA ± SEM.

### Chemokine and cytokine profiling

Cytokine and chemokine profiling were performed using the Bio-Plex Pro™ 40-Plex Human Chemokine Panel (Bio-Rad #171ak99mr2) and the Bio-Plex Pro™ Human Inflammation Panel 1, 37-Plex (Bio-Rad #171AL001M), multiplexed on the same 96-well plate. Cytokine standards were serially diluted and chemokine profiling from all cell challenges was done following manufacturer’s instructions (Bio-Rad) with 4 biological replicates. Quality controls were also included to ensure the validity of the results obtained. The Bio-Plex Manager™ software was used to determine the concentration of the analytes within each sample using the generated standard curves and was expressed in pg/mL. Statistical analysis was done using GraphPad Prism’s one-way analysis of variance (ANOVA) and further analysis was done using Tukey’s multiple comparison test when the ANOVA indicated significant differences were present. All data are shown as the mean pg/mL ± standard error of the mean (SEM). Z-scores were determined and visualized using R version 4.0.0 and package Bioconductor to determine the overall impact of each challenge on cytokine and chemokine production from THP-1 monocytes. To quantify the cytokines produced by PD-THP-1 cells, supernatants were collected from all the treatment conditions mentioned above. IL-1Ra, TGF-β1, IL-10 and IL-8 were detected by following manufacturer protocols of the ELISA kits (R&D Systems, Minneapolis, MN). ELISA plates were read at a 450 nm wavelength using a Synergy HTTR microplate reader (Bio-Tek Instrumentation, VT, USA). Data was collected with the KC4 program for windows (Bio-Tek) and compiled using Microsoft Excel (Microsoft Corp., Redmond, Washington, USA). Each cytokine concentration for the different treatments was generated from a standard curve and expressed in pg/mL. Statistical analysis was done using SigmaPlot (Version 14.5) ANOVA and further analysis was done using Tukey’s multiple comparison test when ANOVA indicated significant differences were present. All data are shown in bar graphs were generated using GraphPad Prism as the mean pg/mL ± SEM to examine for the functional capacity of treatments in PD-THP-1 cells.

### Morphological characterization

Morphological changes in THP-1 human monocytes conditioned with the LrS were determined by staining actin filaments with phalloidin-CF568 (Biotum Inc. Cat. # 00044-T) following manufacturing protocols. PD-THP-1 cells were conditioned with RA and LrS for 48 h, and actin filaments were subsequently stained with phalloidin-CF488 (Biotum Inc. Cat. # 00042-T) following manufacturing protocols. Briefly, following conditioning, cells were fixed with 3.75% formaldehyde, permeabilized with 0.5% Triton X-100, and stained with phalloidin-CF568 (THP-1 cells) and phalloidin-CF488 (PD-THP-1 cells) for 20 min at room temperature. Cells were counter-stained and mounted using ProLong™ Diamond Antifade Mountant with DAPI (ThermoFisher Cat #: P36966).

### Mitochondrial function assay

Mitochondrial activity through mitochondrial substrate utilization was determined using the Mitoplate S-1 system following manufacturer’s protocols (Biolog, Cat. # 14105). THP-1 human monocytes were seeded into a 96-well plate which was pre-coated with dried metabolic substrate probes at a final cell density of 40,000 cells per well. Saponin (100 µg/mL) was used as a permeability agent. Cells were treated with 20% LrS for 24 h and the amount of dye reduction was quantified by measuring the absorbance of the colour change at OD_590_. Fold changes in the utilization of the different metabolic substrates was determined by comparing the absorbance readings of LrS treated cells to an untreated control.

### Analysis of protein lysine acetylation

Protein lysine acetylation in THP-1 human monocytes conditioned with the LrS for 24-, 48-, and 72-h was done using two-dimensional western blot and peptide mass fingerprinting analyses. Cell pellets were harvested following the different conditioning time points, supernatant decanted, and immediately frozen at − 80 °C prior to shipping to Applied Biomics (California, USA) for processing. Anti-acetyl lysine antibodies (Acetylated Lysine Monoclonal Antibody (1C6) Catalog # MA1-2021 from ThermoFisher) were used to detect proteins with acetylated lysine residues and a fold change > 5.0 between the LrS conditioned and corresponding medium controls was chosen as a cut-off for subsequent protein identification using peptide-mass fingerprinting.

### Flow cytometry

Differential cell-surface marker expression of THP-1 s conditioned with the LrS or medium controls was determined using a BD Accuri C6 flow cytometer (Table 4). THP-1 monocytes conditioned with the LrS and PD-THP-1 cells were conditioned with RA, LrS and RA and LrS for 72-h as described above. Media controls were included for all experiments. Following conditioning, 1 × 10^6^ cells were re-suspended in D-phosphate buffered saline (D-PBS) and cells were stained with the viability dye 7-AAD (Tonbo, 13-6993) for 10 min on ice while protected from light. Cells were washed with 1 mL of cell staining buffer (CSB) (4% calf serum, 5 mM EDTA in D-PBS) and centrifuged for 5 min at 400×*g* (4 °C). Immediately following viability staining, non-specific Fc-mediated interactions were blocked by the addition of 100 µL of blocking buffer (10% calf serum (heat inactivated) in D-PBS) to the cell suspension for 10 min. Anti-human FITC CD11c, anti-human APC CD11b, and anti-human PE CD86 or anti-human APC CD64 and anti-human PE CX_3_CR1 were added to the THP-1 cell suspension followed by incubation on ice and protected from light for 30 min. Similarly, anti-human APC CD11b and anti-human PE CD83 or anti-human APC CD64, anti-human FITC CD80 and anti-human PE CD86 or anti-human APC CD103 and anti-human APC CD11c or anti-human PE CD209/DC-SIGN (Dendritic cell-specific intercellular adhesion molecule-3 grabbing non-integrin) were added to the PD-THP-1 cell suspension. Cells were washed with CSB as described above, resuspended in 100 µL of fixation buffer (4% paraformaldehyde-PBS) and incubated for 30 min at room temperature protected from light. Data acquisition was done using the BD Accuri Plus flow cytometer and corresponding software package. 30,000 viable cells (as determined by incorporation of 7-AAD viability dye (Tonbo 13-6993) were acquired for each experiment and subsequent analysis was done using FlowJo v.10 software.

**Table 4:** Antibodies used for flow cytometry analysis.

Name	Manufacturer	Cat. No	Clone	Working concentration
Anti-human APC CD11b	Tonbo	20-0118	ICRF44	0.2 μg/test
Anti-human APC CD11c	Tonbo	20-0116	3.9	0.05 μg/test
Anti-human FITC CD11c	Tonbo	35-0116	3.9	0.2 μg/test
Anti-human APC CD64	BioLegend	305013	10.1	0.04 μg/test
Anti-human APC CD103	BioLegend	350215	Ber-ACT8	0.05 μg/test
Anti-human FITC CD80	BioLegend	305205	2D10	0.08 μg/test
Anti-human PE CD83	BioLegend	305307	HB15e	0.2 μg/test
Anti-human PE CD86	BioLegend	305405	IT2.2	0.02 μg/test
Anti-human PE CD209	BioLegend	330105	9E9A8	0.1 μg/test
Anti-human PE CX_3_CR1	BioLegend	341611	2A9-1	0.02 μg/test

### Supplementary Information

Supplementary Information.

## Data Availability

All relevant data are contained within the article except for the microarray data which has been submitted to the National Center for Biotechnology Information’s Gene Expression Omnibus (https://www.ncbi.nlm.nih.gov/geo/) under accession number GSE225273.

## Abbreviations

APC: Antigen-presenting cells
CCL: Chemokine (C-C motif) ligand
CD: Cluster of differentiation
CXCL: Chemokine (C-X-C motif) ligand
FABP4: Fatty Acid Binding Protein-4
IDH: Isocitrate dehydrogenase
IL-#: Interleukin-#
IL1R2: Interleukin 1 Receptor Type 2
IL4I1: IL-4-induced gene 1
LAB: Lactic acid bacteria
LPS: Lipopolysaccharide
LrS: *Lacticaseibacillus rhamnosus* R0011 secretome
PD-THP-1: PMA-differentiated cells
PGAM1: Phosphoglycerate mutase 1
PMA: Phorbol 12-myristate 13-acetate
PRR: Pattern recognition receptor
RA: Retinoic acid
SOD2: Mitochondrial manganese superoxide dismutase
TDO2: Tryptophan 2,3-dioxygenase
TNFα: Tumor necrosis factor α
