# Supplementary material for: A Lacticaseibacillus rhamnosus secretome induces immunoregulatory transcriptional, functional and immunometabolic signatures in human THP-1 monocytes
Source: Sci Rep. 2024 Apr 10;14:8379. doi: 10.1038/s41598-024-56420-8 (PMC11006683; doi:10.1038/s41598-024-56420-8)
Supplement: Supplementary file 1 — Supplementary Information. [file 41598_2024_56420_MOESM1_ESM.docx]

**Supporting Information**

***Lacticaseibacillus rhamnosus* R0011 secretome induces temporal transcriptional, functional, and immunometabolic signatures in human THP-1 monocytes consistent with M2 immunoregulatory macrophage activity**

Michael P. Jeffrey^1^, Lin Saleem^1, 4^, Chad W. MacPherson^2^, Thomas A. Tompkins^3^, Sandra T. Clarke^4, 1^ & Julia M. Green-Johnson^1^

^1^Applied Bioscience Graduate Program and the Faculty of Science, Ontario Tech University, Oshawa, ON

^2^Lady Davis Institute for Medical Research, Jewish General Hospital, Montreal, QC, H3T 1E2 Canada^.^

^3^Lallemand Bio-Ingredients, Inc., Montreal, QC, Canada.

^4^Guelph Research and Development Centre, Agriculture and Agri-Food Canada, Guelph, ON N1G 5C9, Canada

**Figure S1**. Cytokine profiles from THP-1 human monocytes conditioned with the LrS or L-Lactic Acid matched controls for 24, 48, or 72 hours. Data shown is the mean cytokine production (pg/mL) ± SEM (n = 4). Significant differences between treatments were determined by one-way ANOVA and Tukey’s post-hoc test and are indicated by * *p* < 0.05, ** *p* < 0.01, *** *p* < 0.001, or **** *p* < 0.0001.

**Figure S2**. Chemokine profiles from THP-1 human monocytes conditioned with the LrS or L-Lactic Acid matched controls for 24, 48, or 72 hours. Data shown is the mean chemokine production (pg/mL) ± SEM (n = 4). Significant differences between treatments were determined by one-way ANOVA and Tukey’s post-hoc test and are indicated by * *p* < 0.05, ** *p* < 0.01, *** *p* < 0.001, or **** *p* < 0.0001.

**Figure S3**. Cytokine profiles from THP-1 human monocytes conditioned with the LrS (20% v/v) for 72-hours followed by challenge with LPS (125ng/mL) or LPS alone for 6 hours. Data shown is the mean cytokine production (pg/mL) ± SEM (n = 4). Significance is indicated as * *p* < 0.05, ** *p* < 0.01, *** *p* < 0.001. or **** *p* < 0.0001 as determined by one-way ANOVA and Tukey’s post-hoc test.

**Figure S4**. Chemokine profiles from THP-1 human monocytes conditioned with the LrS (20% v/v) or media control for 72-hours followed by challenge with LPS (125ng/mL) for 6 hours. Data shown is the mean chemokine production (pg/mL) ± SEM (n = 4). Significance is indicated as * *p* < 0.05, ** *p* < 0.01, *** *p* < 0.001. or **** *p* < 0.0001 as determined by one-way ANOVA and Tukey’s post-hoc test.


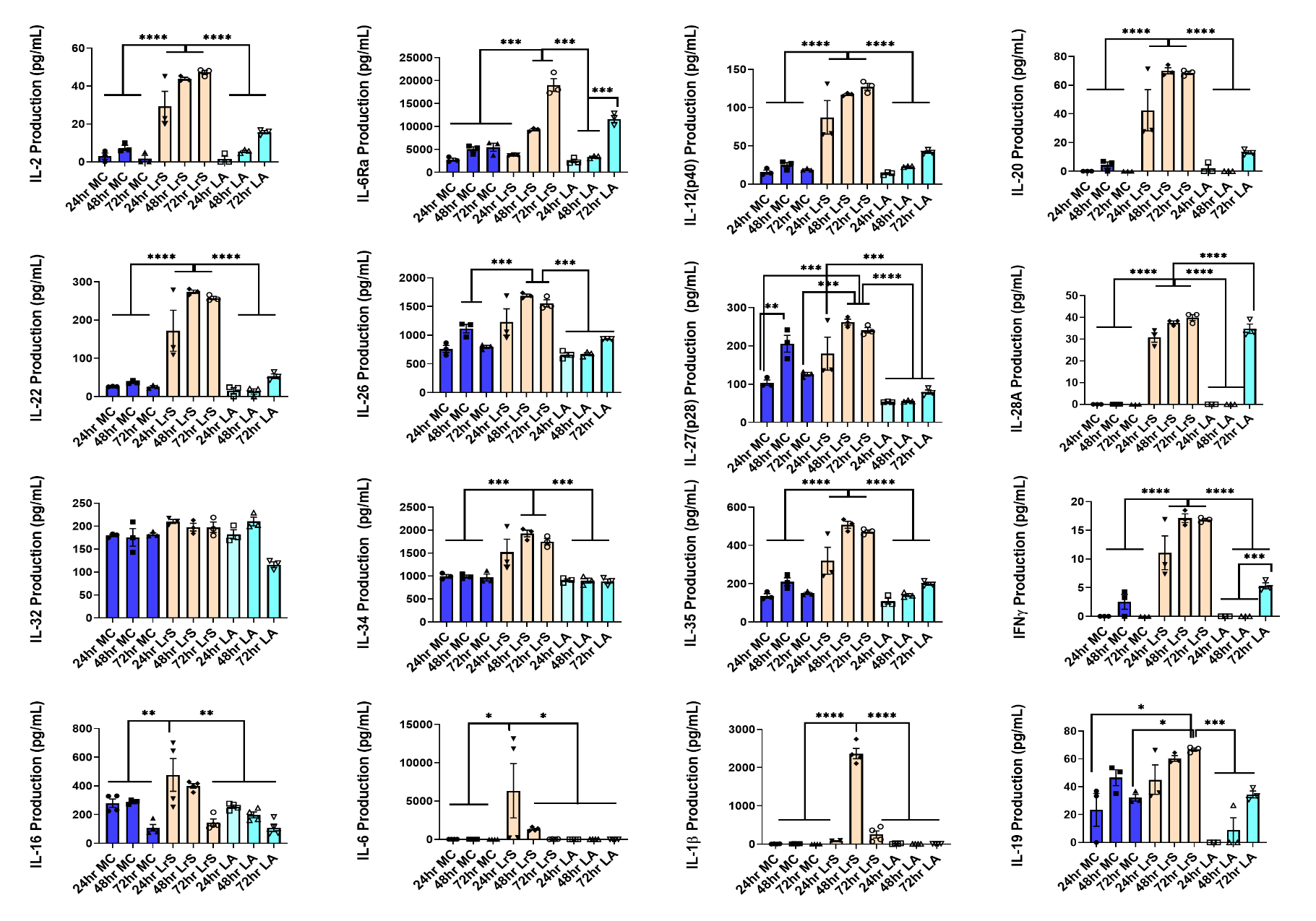


**Figure S1**. Cytokine profiles from THP-1 human monocytes conditioned with the LrS or L-Lactic Acid matched controls for 24, 48, or 72 hours. Data shown is the mean cytokine production (pg/mL) ± SEM (n = 4). Significant differences between treatments were determined by one-way ANOVA and Tukey’s post-hoc test and are indicated by * *p* < 0.05, ** *p* < 0.01, *** *p* < 0.001, or **** *p* < 0.0001.


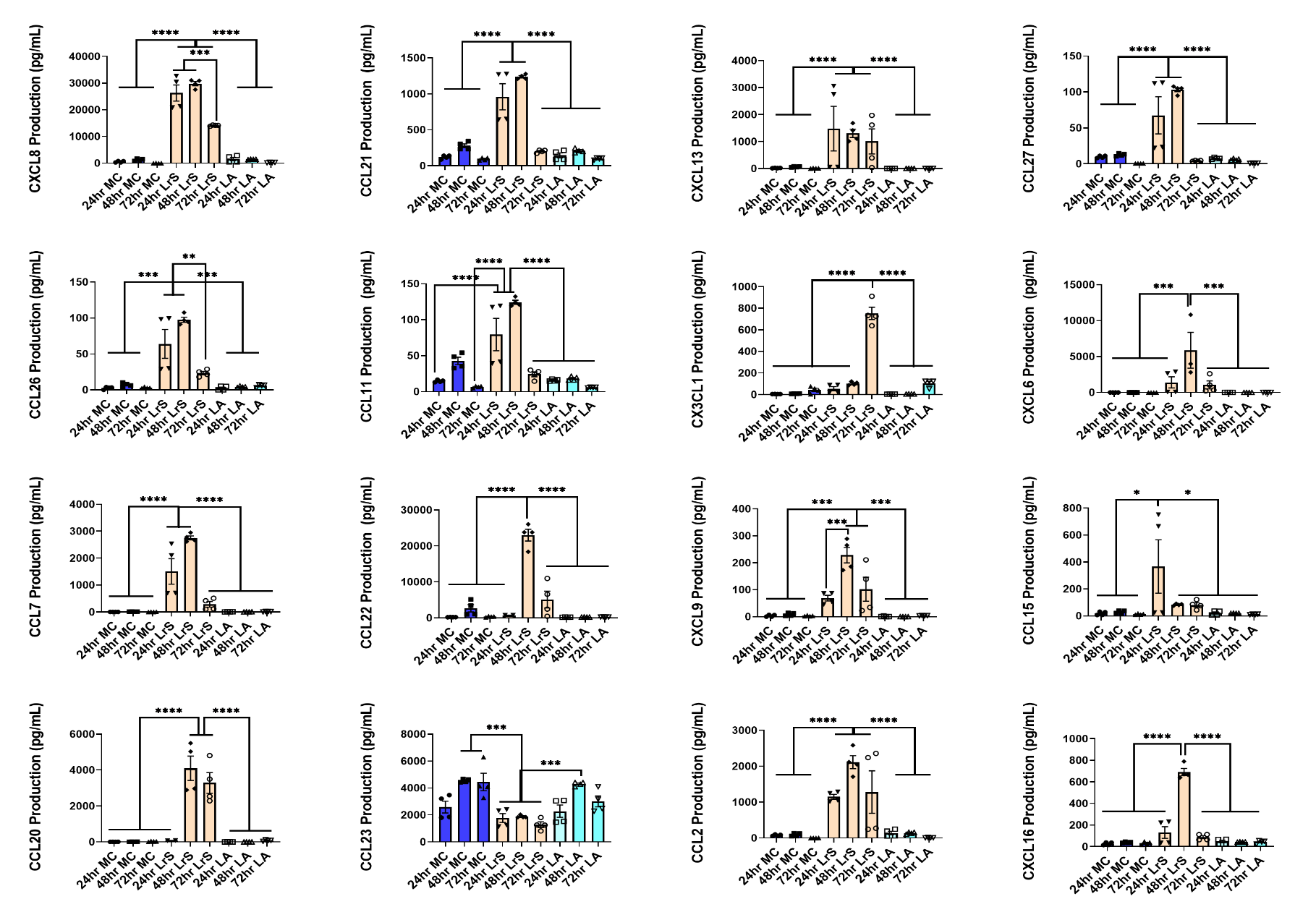


**Figure S2**. Chemokine profiles from THP-1 human monocytes conditioned with the LrS or L-Lactic Acid matched controls for 24, 48, or 72 hours. Data shown is the mean chemokine production (pg/mL) ± SEM (n = 4). Significant differences between treatments were determined by one-way ANOVA and Tukey’s post-hoc test and are indicated by * *p* < 0.05, ** *p* < 0.01, *** *p* < 0.001, or **** *p* < 0.0001.


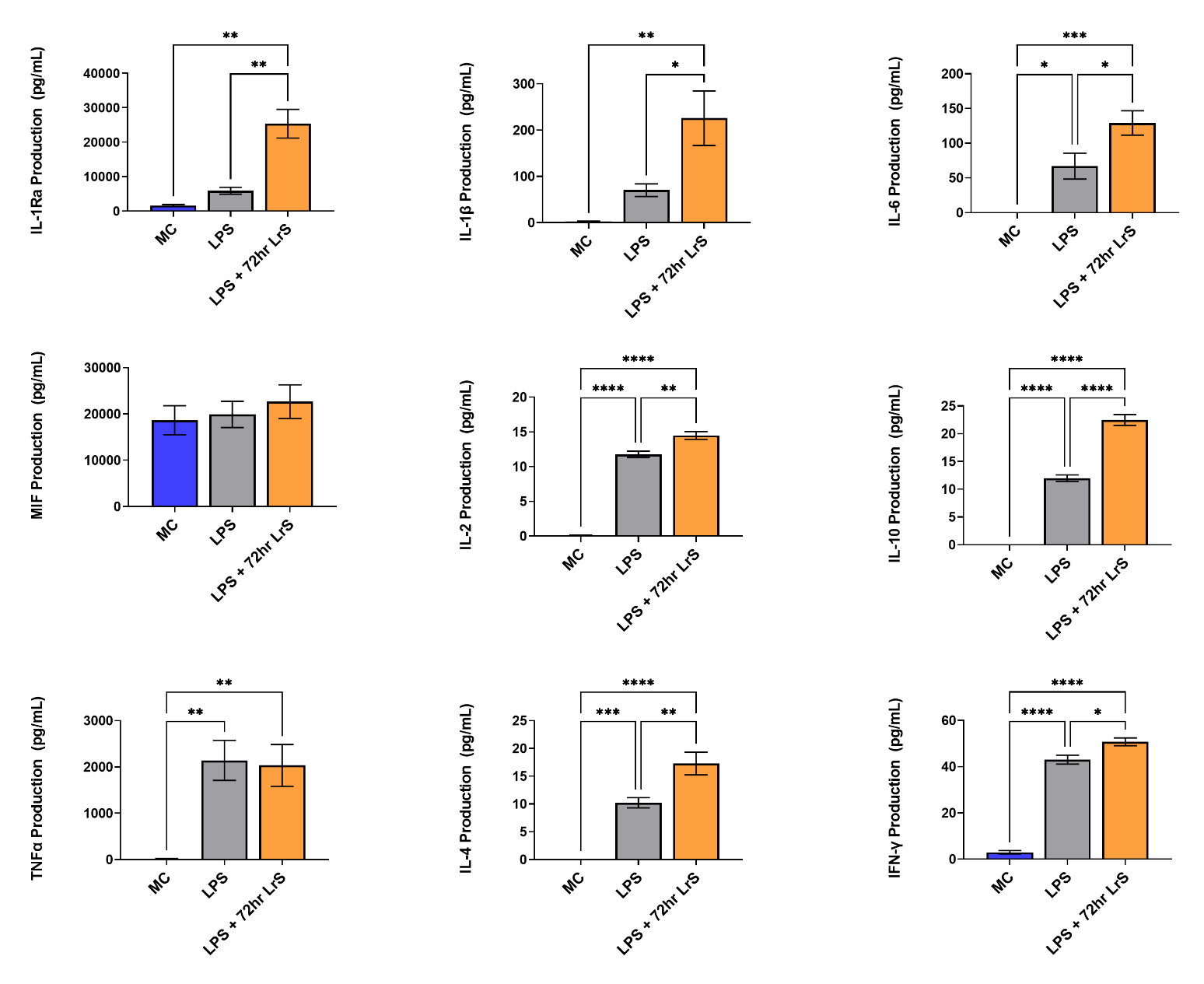


**Figure S3**. Cytokine profiles from THP-1 human monocytes conditioned with the LrS (20% v/v) for 72-hours followed by challenge with LPS (125ng/mL) or LPS alone for 6 hours. Data shown is the mean cytokine production (pg/mL) ± SEM (n = 4). Significance is indicated as * *p* < 0.05, ** *p* < 0.01, *** *p* < 0.001. or **** *p* < 0.0001 as determined by one-way ANOVA and Tukey’s post-hoc test.


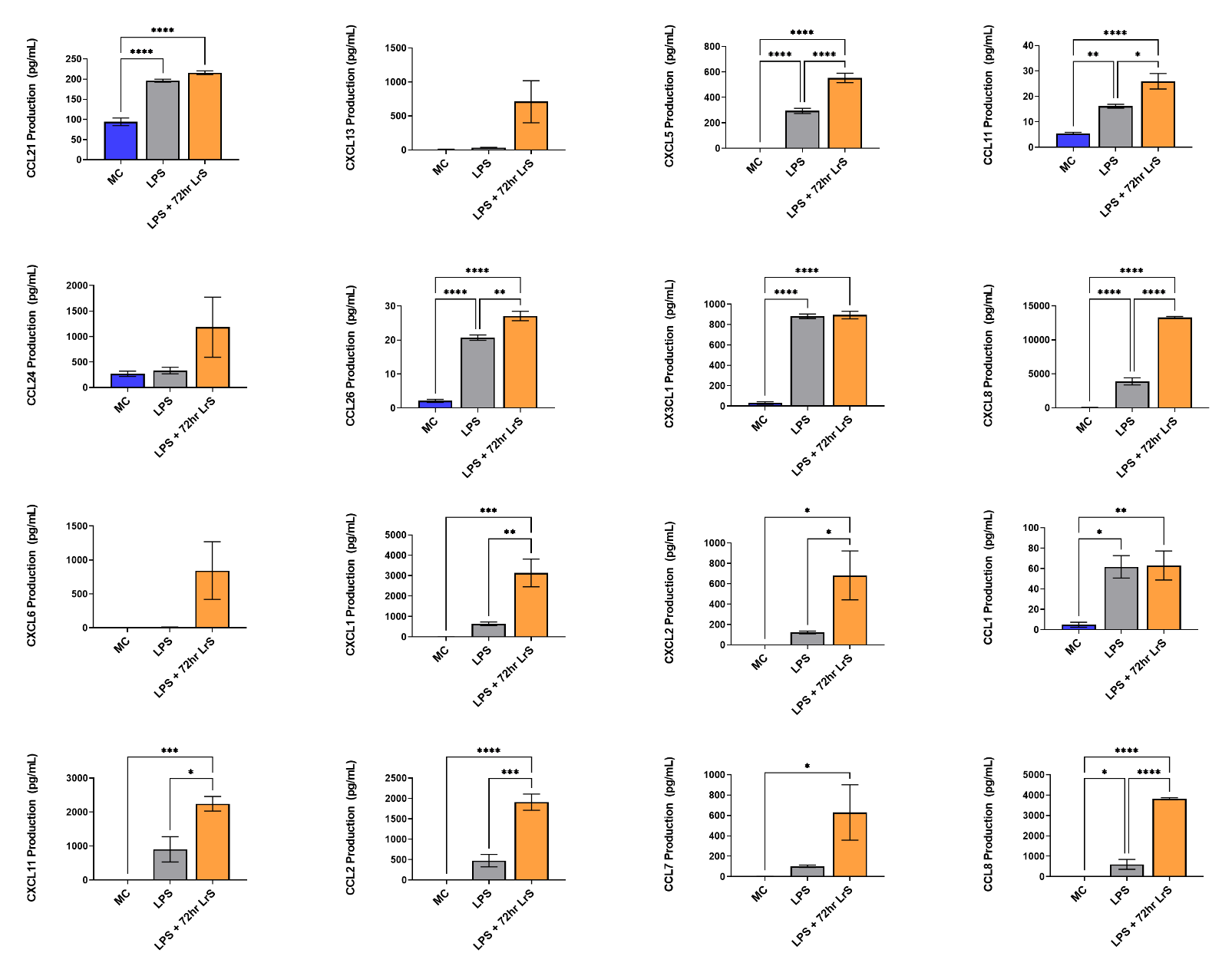


**Figure S4**. Chemokine profiles from THP-1 human monocytes conditioned with the LrS (20% v/v) or media control for 72-hours followed by challenge with LPS (125ng/mL) for 6 hours. Data shown is the mean chemokine production (pg/mL) ± SEM (n = 4). Significance is indicated as * *p* < 0.05, ** *p* < 0.01, *** *p* < 0.001. or **** *p* < 0.0001 as determined by one-way ANOVA and Tukey’s post-hoc test.
